# Dynamically-expressed prion-like proteins form a cuticle in the pharynx of *Caenorhabditis elegans*

**DOI:** 10.1242/bio.20147500

**Published:** 2014-10-31

**Authors:** Julia B. George-Raizen, Keith R. Shockley, Nicholas F. Trojanowski, Annesia L. Lamb, David M. Raizen

**Affiliations:** 1 Department of Neurology, Perelman School of Medicine, University of Pennsylvania, Philadelphia, PA 19104, USA; 2 Biostatistics Branch, National Institute of Environmental Health Sciences, National Institutes of Health, Department of Health and Human Services, Research Triangle Park, NC 27709, USA; 3 Present address: Department of Earth and Environmental Sciences, The Graduate Center, The City University of New York, 365 Fifth Avenue, New York, NY 10016, USA.

**Keywords:** *C. elegans*, molting, larvae, amyloid, cuticle, ABU/PQN, innate immunity, unfolded protein response

## Abstract

In molting animals, a cuticular extracellular matrix forms the first barrier to infection and other environmental insults. In the nematode *Caenorhabditis elegans* there are two types of cuticle: a well-studied collagenous cuticle lines the body, and a poorly-understood chitinous cuticle lines the pharynx. In the posterior end of the pharynx is the grinder, a tooth-like cuticular specialization that crushes food prior to transport to the intestine for digestion. We here show that the grinder increases in size only during the molt. To gain molecular insight into the structure of the grinder and pharyngeal cuticle, we performed a microarray analysis to identify mRNAs increased during the molt. We found strong transcriptional induction during the molt of 12 of 15 previously identified *abu* genes encoding Prion-like (P) glutamine (Q) and asparagine (N) rich PQN proteins, as well as 15 additional genes encoding closely related PQN proteins. *abu/pqn* genes, which we name the *abu/pqn* paralog group (APPG) genes, were expressed in pharyngeal cells and the proteins encoded by two APPG genes we tested localized to the pharyngeal cuticle. Deleting the APPG gene *abu-14* caused abnormal pharyngeal cuticular structures and knocking down other APPG genes resulted in abnormal cuticular function. We propose that APPG proteins promote the assembly and function of a unique cuticular structure. The strong developmental regulation of the APPG genes raises the possibility that such genes would be identified in transcriptional profiling experiments in which the animals' developmental stage is not precisely staged.

## INTRODUCTION

Ecdysozoa, the clade of animals defined by the presence of an exoskeleton, exchange their cuticle during episodic molts (Aguinaldo et al., 1997; Dunn et al., 2008). Ecdysozoan species that have a flexible exoskeleton grow in a continuous fashion while species with rigid exoskeletons grow in a saltatory fashion, with the animals enlarging only during the molt. The nematode *C. elegans* shows both growth modes. The body, which is lined by an elastic collagenous cuticle, grows continuously, whereas the buccal cavity, which lines the entrance to the pharynx and contains rigid chitin (Veronico et al., 2001), grows in a saltatory fashion (Knight et al., 2002). The grinder, a cuticular specialization in the posterior end of the pharynx, macerates the animal's food (bacteria) prior to transport to the intestine. Protein components of the pharyngeal cuticle, the grinder, and buccal cuticle have not been defined. To gain molecular insight into the structure of the buccal cavity, pharyngeal cuticle, and grinder, we performed a transcriptional profiling experiment in precisely staged molting and non-molting larvae. Our data led to the identification of proteins we term the ABU/PQN Paralog Group (APPG) proteins, as components of the pharyngeal cuticle. In addition, our results call for a re-interpretation of prior observations related to some of the APPG genes.

## RESULTS

### The pharyngeal grinder grows in a saltatory fashion

**Fig. 1.**
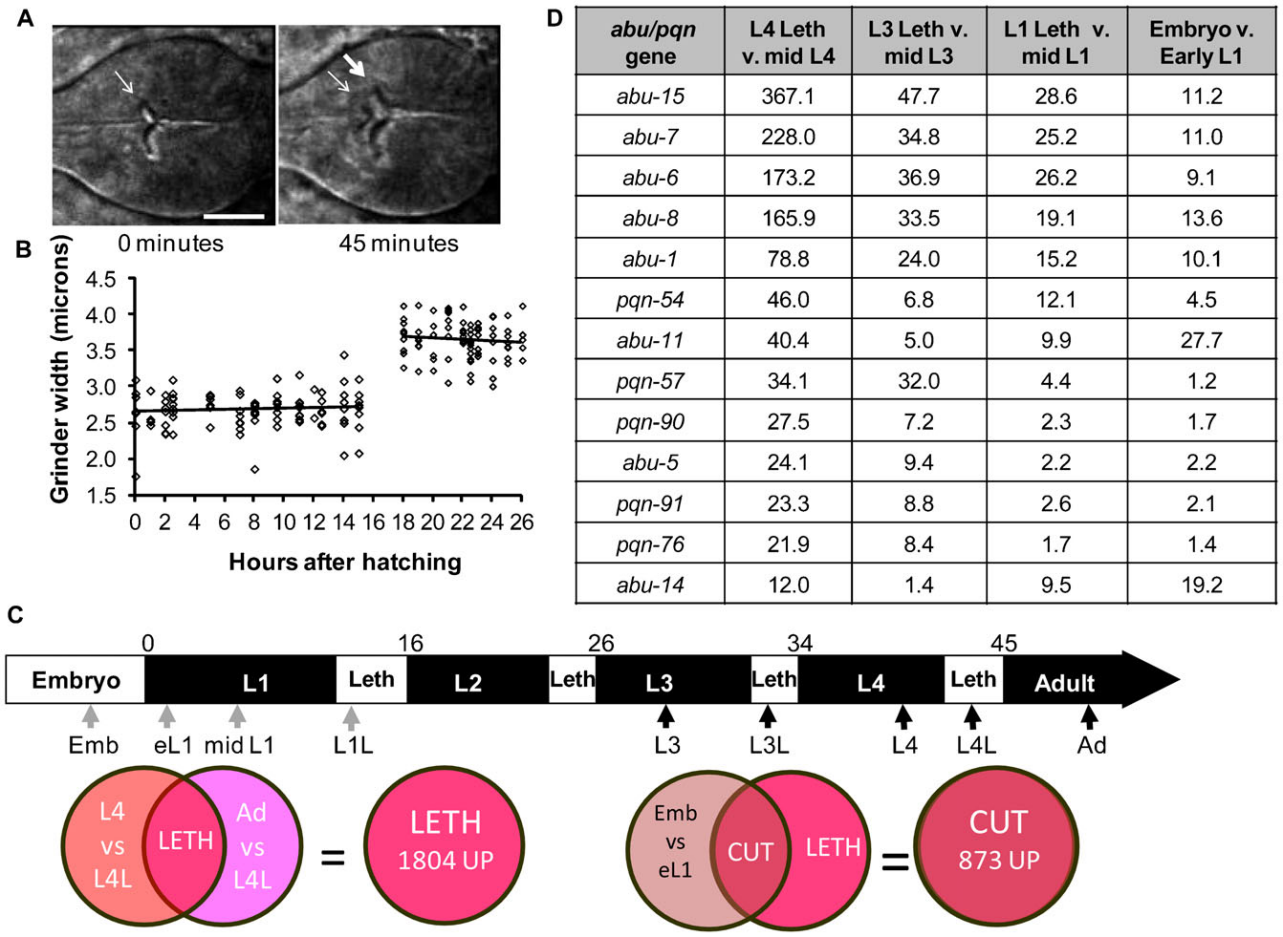
*abu/pqn* paralog group genes are up-regulated during cuticle synthesis. (A) Pictures of a wild-type posterior pharynx at the start (0 minutes after pumping cessation) and middle (45 minutes after pumping cessation) of the fourth larval stage (L4) lethargus period. Thin and thick arrows denote the L4 and adult grinders, respectively. Anterior is to the left. Scale bar is 10 µm. (B) Width of the grinder as a function of time after hatching. First larval stage (L1) was 0–15 hours after hatching and the second larval stage was 18–26 hours after hatching. L1 lethargus occurred between 15 and 18 hours after hatching. Each point corresponds to one worm. Linear regression of the data within each larval stage produced lines with slopes that were not significantly different from zero (one-way ANOVA, p>0.1). (C) Illustration of *C. elegans* larval development. L1–L4 denotes larval stages 1–4, and Leth denotes lethargus. Numbers above the figure denote hours after hatching. Arrows mark sampling times for RNA collections. Dark arrows correspond to samples collected in this study whereas gray arrows correspond to sampling collected in Baugh at el. (Baugh et al., 2009). Venn diagrams illustrate logic used to define the L4 lethargus gene set and the Cuticle gene set. (D) The 13 most highly-expressed *abu/pqn* genes identified in all stages of cuticular synthesis. Shown is the fold change in the four comparisons. Additional genes in the *abu/pqn* paralog group are listed in supplementary material Table S5.

The buccal cavity cuticle, which lines the entrance to the pharynx, grows only during the molts, much like the body cuticle of arthropods (Knight et al., 2002). We wondered whether this was also true of other parts of the pharyngeal cuticle, such as the grinder. Time lapse analysis of an animal in fourth larval stage (L4) molt showed anterior movement of the L4 grinder followed by formation of the adult grinder posterior to the L4 grinder (Fig. 1A; supplementary material Movie 1). The posterior, new grinder was larger (Fig. 1A), suggesting that the grinder too grows in a saltatory fashion. To test this suggestion, we measured grinder size during the first two larval stages. The grinder stayed a constant size within each larval stage and enlarged only during the molt (Fig. 1B). These data suggested that genes involved in grinder synthesis would be induced specifically during the molt.

### APPG genes are strongly induced during cuticle synthesis periods

The pharyngeal cuticle, buccal cavity, and grinder are synthesized 5 times during *C. elegans* life cycle: during late embryogenesis prior to hatching and during each of the four larval transition stages as the animal develops to adulthood. The larval transition stage is called lethargus, a sleep-like state defined by a cessation of feeding (Cassada and Russell, 1975; Singh and Sulston, 1978; Raizen et al., 2008). To identify genes differentially expressed during the molt, we collected RNA at precise time points relative to the fourth larval stage (L4) lethargus: (1) approximately three hours prior to L4 lethargus; (2) 30–40 minutes after feeding cessation, which marks the start of L4 lethargus (Fig. 1C); and (3) in the pre-reproductive young adult stage, four hours after the end of L4 lethargus (Fig. 1C). To identify genes expressed during other molts we collected RNA from the third larval stage (L3) lethargus as well as from the mid L3 stage (Fig. 1C).

1804 gene transcripts were up regulated (supplementary material Table S1) and 1088 gene transcripts were down regulated (supplementary material Table S2) during the L4 lethargus period compared with flanking time periods, (late-L4 and young adult stages) at a 5% false discovery rate. To distinguish genes associated with lethargus behavior or escape from the prior stage cuticle from those associated with synthesis of the cuticle, we compared our L4 lethargus-enriched gene set with a published microarray dataset of genes induced in the late embryonic stage prior to hatching (Baugh et al., 2009). Animals at this stage synthesize their first cuticle, but do not display lethargus behavior or escape from a cuticle (Sulston et al., 1983). We define this gene set, which contains 873 up-regulated (supplementary material Table S3) and 686 down-regulated gene transcripts (supplementary material Table S4), as the Cuticle gene set (Fig. 1C).

Many of the genes that had the highest fold changes in the cuticle gene set encode proteins that were originally identified by their amino acid composition. These glutamine- and asparagine-rich proteins have been predicted to form prions and have been named PQN proteins (Michelitsch and Weissman, 2000). Of the 107 *pqn* genes encoded in the genome, 15 have been renamed *abu* (Urano et al., 2002; Sun et al., 2011). We refer to 12 of the *abu* genes and 17 additional genes that encode proteins with closely related amino acid sequences as the *abu/pqn* paralog group (APPG) genes. These 29 proteins are more similar to each other than to other PQN proteins (supplementary material Fig. S1). Features distinguishing the APPG proteins from other PQN proteins are a high percentage of cysteines (≥9%), and the presence of one or more repeat CxxxCxxxC (PF02363: C_tripleX) (supplementary material Fig. S2). In addition, 27 of the 29 proteins contain predicted signal sequences (supplementary material Fig. S2), suggesting that they are secreted. APPG genes also share enriched expression in cuticular synthesis periods (Fig. 1D; supplementary material Fig. S1; Table S5). Of 27 APPG genes represented on the array, 26 were up regulated during one or more cuticular synthesis period. In contrast, only eight of the remaining 74 *pqn* genes represented on the array were up regulated during the same intervals (left side of supplementary material Fig. S1).

### APPG genes are expressed in pharyngeal muscle cells and two APPG proteins localize to the pharyngeal cuticle

**Fig. 2.**
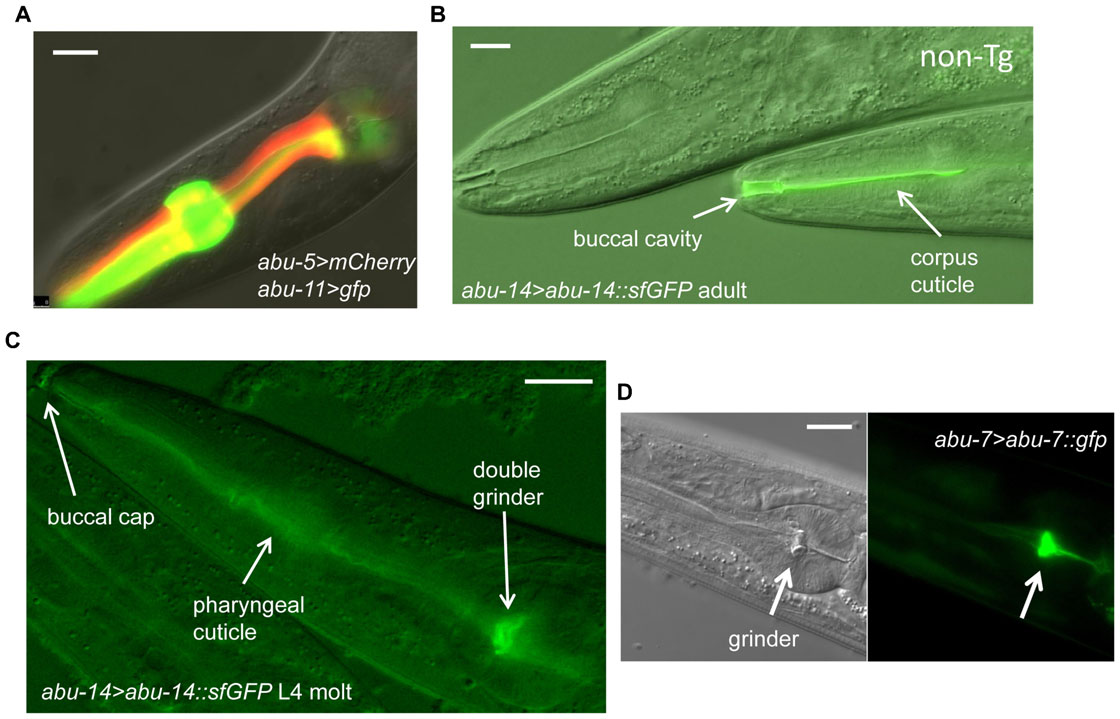
*abu/pqn* genes are expressed in the pharyngeal cuticle. (A) Expression of *abu-5* (red) and *abu-11* (green) in pharyngeal cells. Scale bar is 10 µm. (B) Differential interference contrast (DIC) and fluorescence image of the anterior pharynx of an adult transgenic animal containing the transgene *abu-14>abu-14::sfGFP*. The fluorescence localizes to the pharyngeal corpus and buccal cavity cuticle (arrows). A non-transgenic sister (marked with “non-Tg”) shows no fluorescence. Anterior is to the left. Scale bar is 10 µm. (C) DIC and fluorescence image of the pharynx of a fourth larval stage molting animal containing the transgene *abu-14>abu-14::sfGFP*. Green fluorescence is seen in both the L4 and adult pharyngeal grinders, in the pharyngeal cuticle, and in the buccal cap. Anterior is to the left. Scale bar is 10 µm. (D) DIC (left) and fluorescence (right) images of a larval animal containing the transgene *abu-7>abu-7::GFP*. The pharyngeal grinder is brightly fluorescent. Scale bar is 10 µm.

Fluorescent transcriptional reporters of all APPG genes we tested (*abu-5, abu-6, abu-11, abu-14, abu-15, pqn-57*) were expressed in pharyngeal muscle (Fig. 2; supplementary material Fig. S3), suggesting that most or all of the APPG genes are expressed in pharyngeal muscle. This suggestion is supported by prior reporter transgene expression analysis of *abu-1* (Urano et al., 2002) and *abu-14* (Ao et al., 2004) and by RNA *in situ* hybridization analysis of *abu-8*, *abu-14*, and *pqn-13* expression (http://nematode.lab.nig.ac.jp), all of which have been demonstrated to be expressed in pharyngeal muscle. In rare cases, expression was seen outside the pharynx: the *pqn-57* reporter was also expressed near the rectum (supplementary material Fig. S3) and RNA in situ analysis showed rare *abu-14* staining in the intestine (http://nematode.lab.nig.ac.jp). Consistent with the microarray data, indicating dynamic expression of APPG genes and a role during the molt, we observed dynamic expression of a fluorescent transcriptional reporter for *abu-11* (supplementary material Fig. S4). Consistent with a larval-specific function of APPG genes, transcriptional reporters for *abu-5*, *abu-11*, *abu-14*, and *abu-15* showed little to no adult fluorescence (supplementary material Fig. S5). In contrast, expression of a *pqn-57* reporter persisted in adult pharynxes (supplementary material Fig. S5).

To determine where in the pharynx the APPG gene products are localized, we studied the expression of fusion proteins between ABU/PQN and green fluorescent protein (GFP). The transgene expressing the ABU-14::GFP fusion protein was able to partially substitute for the function of the *abu-14* gene (see below), suggesting that the localization of the green fluorescence reports the localization of the endogenous ABU-14 protein. We used superfolder GFP (sfGFP) (Pédelacq et al., 2006) to allow us to see fluorescence at low concentrations of injected transgene, as we found that high concentrations were toxic (see below).

We observed ABU-14::sfGFP fluorescence in or near the pharyngeal cuticle and in the cuticle lining the buccal cavity (Fig. 2B). To determine whether ABU-14 was in the cuticle rather than near the pharyngeal cuticle, we examined animals undergoing the L4 molt. Late in the molt, both the smaller anterior grinder and the larger posterior grinder showed green fluorescence in transgenic animals expressing ABU-14::sfGFP fusion proteins (Fig. 2C). In addition, the buccal cap at the anterior nose of the animal was also fluorescent (Fig. 2C), indicating that it represents extruded pharyngeal cuticle material.

To determine if other APPG proteins are localized to the grinder and pharyngeal cuticle, we also examined the localization of ABU-7::GFP. As in the case of ABU-14 reporter, we observed ABU-7 localization in the pharyngeal grinder (Fig. 2C), though we do not yet know whether this transgene is functional.

### APPG genes *abu-6* and *abu-14* are required for the structure and function of the pharyngeal cuticle

**Fig. 3.**
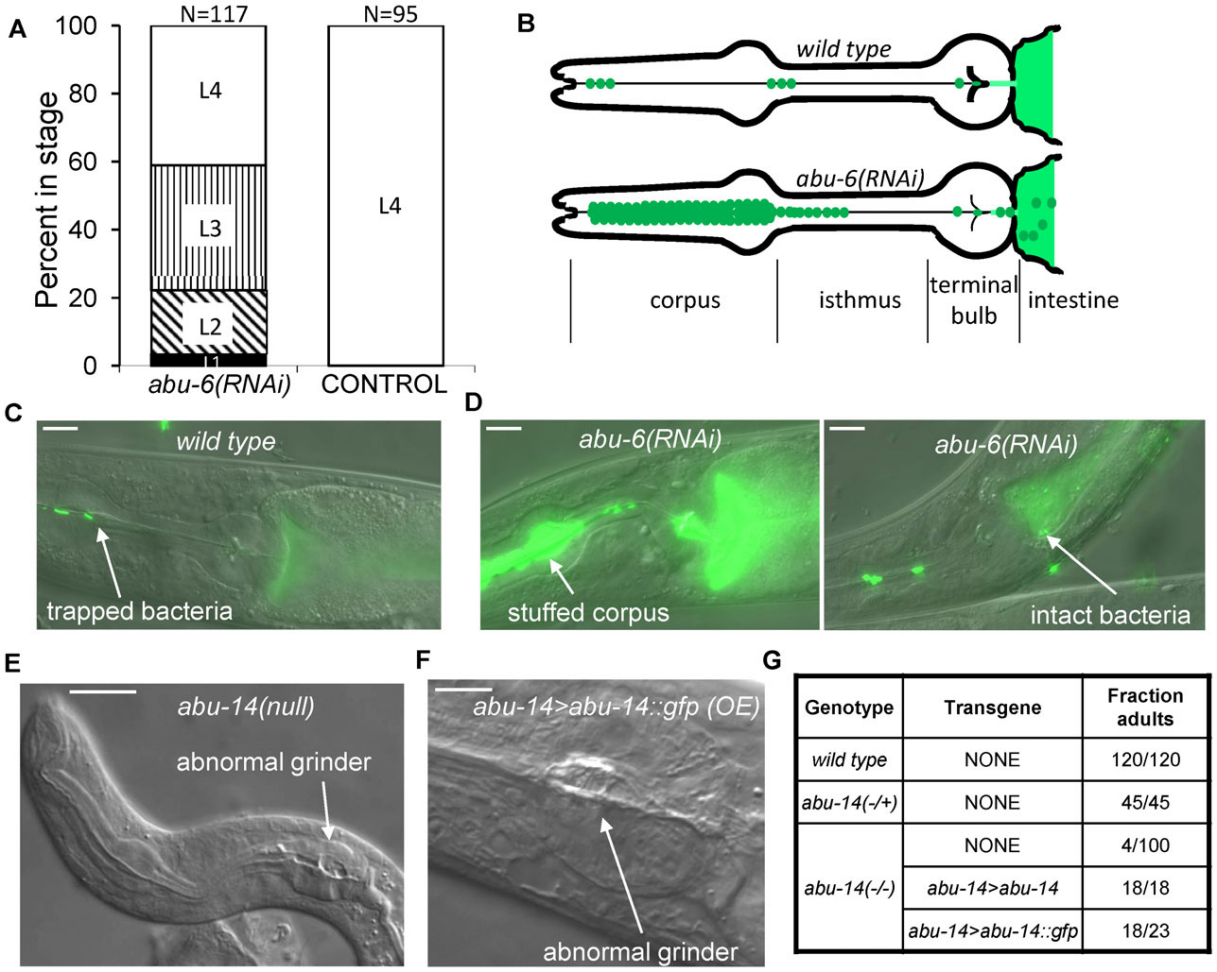
Disruption of *abu/pqn* gene function results in cuticular abnormalities in the pharynx and poor digestion of bacteria. (A) Developmental delay of transgenic animals expressing *myo-2>abu-6(RNAi)* in comparison to control transgenic animals expressing *abu-5>mCherry*. White, vertical, diagonal, and black bars denote, L4, L3, L2 and L1 stages, respectively. (B) Cartoon depicting observations of feeding in wild-type animals and in *abu-6(RNAi)* animals. Normally, bacteria (green) are trapped in the corpus and anterior isthmus, and are disrupted by the grinder in the terminal bulb to release their contents into the anterior intestine (lighter color green). In *abu-6(RNAi)* transgenic animals, bacteria accumulate in the corpus and are poorly disrupted by the grinder, resulting in the presence of intact bacteria in the anterior intestine. (C) Example of a wild-type animal with bacteria trapped in the anterior isthmus and with disrupted green bacteria in the anterior intestine. Anterior is to the left. Scale bar is 10 µm. (D) *abu-6(RNAi)* animals fed fluorescent bacteria have a pharyngeal corpus stuffed with bacteria as well as intact bacteria in the anterior intestine. Anterior is to the left. Scale bar is 10 µm. (E) Aberrant pharyngeal development observed in an *abu-14(ok1789)* arrested first larval stage animal. Arrow indicates abnormal grinder. Anterior is to the left. Scale bar is 10 µm. (F) Aberrant pharyngeal cuticle in an adult animal over-expressing *abu-14::gfp*. Arrow indicates an abnormal grinder. Anterior is to the left. Scale bar is 5 µm. (G) In the absence of *abu-14*, most animals do not reach adulthood 3 days after hatching. This defect is fully rescued by a genomic DNA fragment containing the *abu-14* gene and is partially rescued by a DNA fragment encoding an ABU-14::GFP protein fusion expressed under the control of an *abu-14* promoter.

To determine the consequence of reduction of APPG gene function, we initially used an RNA interference knock-down approach. Given the high similarity between APPG proteins, we reasoned that there might be a high degree of degeneracy in their function and that, in order to detect a phenotype, knocking down more than one of the APPG genes would be required. We generated transgenic animals in which pharyngeal muscle cells expressed both the sense and anti-sense strands of *abu-6*. Because of the high nucleotide sequence identity among APPG genes, this RNAi treatment is predicted to affect several APPG genes (supplementary material Table S6). Transgenic animals grew poorly (Fig. 3A), likely because of inefficient pharyngeal transport and grinding of bacteria (Fig. 3B). This was evident by the high frequency of animals with their pharyngeal lumen stuffed with bacteria as well as occurrence of undigested bacteria in the anterior intestine (Fig. 3D). Whereas 0/50 wild-type animals had a stuffed pharynx phenotype or intact GFP-marked bacteria in the anterior intestine, 19/32 *abu-6(RNAi)* animals had a stuffed pharynx phenotype and 6/32 had intact bacteria in the anterior intestine. These results indicate that APPG protein function is required for the digestive action of the pharynx, consistent with the expression pattern.

During the course of our transgenic RNAi experiments, the deletion allele *ok1789* became available for the gene *abu-14*. In *abu-14(ok1789)* mutants, the protein's signal sequence is eliminated, suggesting that the protein is non-functional. 96% of *abu-14(ok1789)* mutants arrested during development as early larvae (Fig. 3E,G). These arrested larvae displayed aberrant pharyngeal grinders, and, in rare cases, lacked a grinder completely (Fig. 3E). The rare *abu-14(ok1789)* homozygous animals that reached adulthood were small and, like the *abu-6(RNAi)* treated animals, failed to fully disrupt bacteria and accumulated bacteria in the pharyngeal lumen. They were also sterile. The larval arrest and poor bacterial digestion phenotypes but not the sterility were rescued by transgenic expression of a 4.7 kb genomic DNA fragment that contained the *abu-14* coding sequences and 3′-untranslated region (UTR) (Fig. 3G) as well as upstream DNA elements. These phenotypes were also rescued by expressing the same construct with GFP and a heterologous 3′-UTR substituting for the *abu-14* stop codon and 3′-UTR (Fig. 3G). Together, these data indicate that APPG genes function in the pharynx and are required for normal pharyngeal cuticular structure and function.

We noted that transgenic animals generated by injection of a high concentration (50 mg/liter) of the ABU-14::GFP transgene were small and pale, and often arrested as young larva or required over a week to reach adulthood (in comparison to two days for wild-type animals). Microscopic analysis showed that some of these animals had deformed grinders (Fig. 3F). These observations further support the idea that ABU-14 is involved in the formation of the pharyngeal grinder. While the GFP moiety of the expressed transgene may be affecting ABU-14 protein function, another possibility is that because these APPG proteins may be interacting to form a complex protein structure, there are stoichiometric constraints on protein expression levels.

### APPG genes are not activated by a blocked unfolded protein response or by infection

**Fig. 4.**
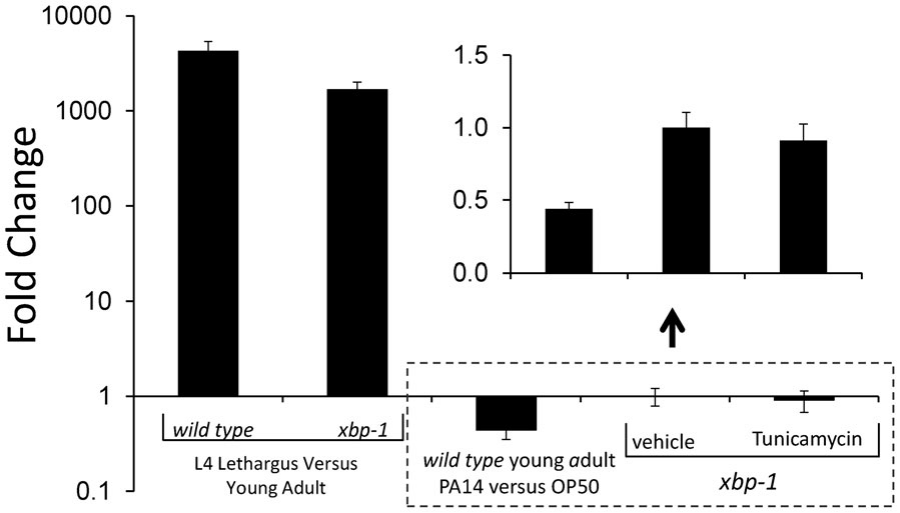
*abu/pqn* genes are induced during L4 lethargus but are not induced by a blocked unfolded protein response or in response to *Pseudomonas aeruginosa* strain PA14 infection. Quantitative reverse transcriptase polymerase chain reaction analysis using oligonucleotide primers that amplify *abu-6*, *abu-7*, *abu-8,* and *abu-15*. The average of four biological replicates is shown on a logarithmic scale. Error bars denote standard deviation. There is strong induction of *abu-6, -7, -8* and *-15* gene expression in L4 lethargus in comparison to 4-hour old adults in both wild-type and *xbp-1* genetic backgrounds, but no induction in young adults in response to PA14 exposure. Treatment of *xbp-1* young adults with Tunicamycin or with the vehicle 0.5% DMSO does not result in *abu-6*, *-7*, *-8* and *-15* gene induction. Inset shows on a magnified non-logarithmic *y*-axis the results of the three conditions bounded by the dotted box (the *x*-axis is at *y* = 1 on the logarithmic plot and *y* = 0 in the inset.).

The subset of the PQN proteins that were renamed ABU have been described as being “Activated by a Blocked Unfolded protein response” (Urano et al., 2002; Sun et al., 2011). This name is based on the observation of increased expression in response to ER stress in animals mutant for *xbp-1*, which is required for the canonical unfolded protein response. Induction of these same *abu* genes has been observed with a number of other perturbations (Viswanathan et al., 2005; Haskins et al., 2008; Mair et al., 2011; Sun et al., 2011; Sahu et al., 2012). The degree of transcriptional induction of the *abu* genes in those experiments was modest, in some cases between 1.5-fold and 2.0-fold (see supplementary material Table S7 for complete comparison). The strong developmental regulation we observe of the APPG genes and the high levels of expression suggested to us that inadvertent inclusion of small numbers of molting animals is sufficient to account for the reported observations. For instance, 0.5% enrichment in L4 lethargus animals in a population that is otherwise made up of young adults or of mid L4 animals would result in an overall approximately two-fold higher expression of *abu-7*. Indeed, statistical comparisons of the published data sets reveal significant overlap between these *abu*-containing gene sets and our L4L gene set (supplementary material Table S8). Notably, the overlap extends to non-APPG genes also induced by these perturbations (examples given in supplementary material Table S7). While we observed strong *abu* gene induction during the molt in both wild-type animals and *xbp-1* mutants (Fig. 4), we did not observe the previously reported induction of four *abu* genes in *xbp-1* mutant young adult animals that were carefully staged to avoid the L4 molt (Fig. 4). Similarly, we did not observe the reported induction of *abu* gene expression with exposure of young adult animals to the pathogenic *Pseudomonas aeruginosa* bacterial strain PA14 (Fig. 4).

Since some of the studies identifying APPG genes as induced by a blocked unfolded protein response reported the harvesting of larval animals (Sun et al., 2011), we repeated the ER stress experiment in larvae. We performed this experiment in first larval stage (L1) animals because, among the four larval stages, the L1 larval period is the only one sufficiently long (16 hours) to allow a five-hour exposure to Tunicamycin without the risk of harvesting molting or nearly molting animals. Beginning three hours after initiation of larval development, we exposed *xbp-1* mutant animals to Tunicamycin for five hours. We collected RNA from these animals as well as from control animals eight hours after initiation of larval development. qPCR analysis showed no difference in expression of the four APPG genes tested (1.05±0.23-fold induction with Tunicamycin, p>0.2, Student's two-tailed t test, N = 3 biological samples for each condition).

### Amyloid dyes stain the pharyngeal cuticle

**Fig. 5.**
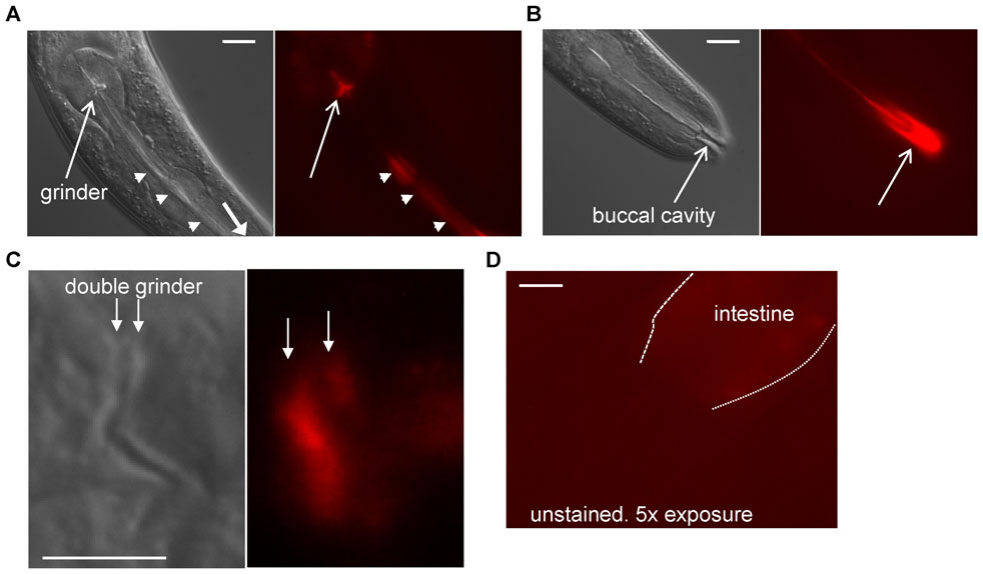
Pharyngeal cuticle stains with amyloid dye Congo Red. (A) DIC (left) and fluorescence (right) images that focus on the grinder (arrow) and pharyngeal lumen (arrow heads) of an animal stained with Congo Red. Anterior is down and to the right. Scale bar is 10 µm. (B) DIC (left) and fluorescence (right) images that focus on the buccal cavity (arrow) of the same animal shown in panel A. Scale bar is 10 µm. (C) DIC (left) and fluorescence (right) images that focus on the double grinder during the L4 molt. Both grinders (marked with arrows) stain with Congo Red. Anterior is to the left. Scale bar is 5 µm. (D) Camera exposure times 5 times greater than those used in panel C show no grinder fluorescence in unstained animals. Anterior is to the left and down. Scale bar is 10 µm.

The PQN proteins are predicted to form amyloid (Michelitsch and Weissman, 2000), which would have the rigid and protease-resistant properties desired for the grinder and pharyngeal lining. Based on the prediction that APPG proteins form amyloid, we stained animals with the amyloid dye Congo Red. The dye stained the pharyngeal lumen, grinder, and buccal cavity (Fig. 5A,B), supporting the notion that the pharyngeal cuticle has amyloid properties. Close observation of stained molting animals with two grinders showed that both grinders stained with Congo Red (Fig. 5C), indicating that it is likely the grinder material itself that stains with the dye. The red staining was not simply autofluorescence of the cuticle since prolonged camera exposures revealed no red signal in the grinders of animals that had not been stained with Congo Red (Fig. 5D). Consistent with these observations, a prior study focusing on pathogenic amyloid deposition noted incidental staining of the buccal cavity with the amyloid dye X-34 (Link et al., 2001). We also stained *abu-14(ok1789)* mutants with Congo Red. With exception of the animals that lacked a grinder (2 animals out of 35 examined), which also lacked Congo Red staining in the terminal bulb, these mutants showed Congo Red staining of pharyngeal cuticle similar to wild-type animals.

## DISCUSSION

### Construction and deconstruction of the pharyngeal cuticle during larval molts

In this report we provide molecular insight into the developmental changes that occur to the *C. elegans* pharyngeal cuticle during larval development. Knight et al. demonstrated that the *C. elegans* buccal cavity cuticle, which is located at the anterior end of the pharynx, grows in bursts during larval development (Knight et al., 2002). We here show that this type of growth also occurs for a cuticular structure at the posterior end of the pharynx, the grinder.

The process of grinder replacement at the molt is carefully orchestrated. It begins with the anterior displacement of the older grinder. The assembly of the new grinder and the dissolution of the older grinder then occur simultaneously or nearly simultaneously. In light of these observations, a potential explanation for the absence of pharyngeal pumping during lethargus periods may be to allow the delicate assembly of the new grinder. Alternatively, the pharynx may be diverting energetic resources used for excitable cell function between molts to the construction of the new cuticle during the molt (see more below).

### Dynamic expression of APPG proteins during larval development

We began these experiments looking for genes that were differentially expressed during the L4 lethargus in comparison to flanking periods. Animals were selected for analysis based on strict behavioral criteria. Having identified 1804 genes that were up-regulated (as well as 1088 that were down-regulated) we then refined the gene list by comparing to genes induced during other cuticle synthesis periods. Most of the APPG genes were identified in each of our 4 microarray sampling periods (supplementary material Fig. S1), leading to our conclusion that their expression is highly dynamic during larval development.

Three recent publications support our conclusion that the APPG genes show dynamic expression during larval development. Hendriks and colleagues sampled mRNA hourly for a period spanning the L3 to the adult stages of *C. elegans* (Hendriks et al., 2014). All 15 of the APPG genes detected by their RNA-sequencing method showed significant cycling of expression. Consistent with our results, they found that expression of these APPG genes was temporally clustered (Hendriks et al., 2014). Kim and colleagues performed RNA sequencing on *C. elegans* larval samples collected every two hours from zero to 48 hours after initiation of larval development (Kim, D., et al., 2013). They identified 18 APPG genes with oscillating expression levels. They too observed temporal clustering of these genes. Finally, Snoek et al. performed a microarray analysis on samples collected hourly from *C. elegans* cultures starting with the late L3 stage and ending with early adult stage (Snoek et al., 2014). Of the 27 APPG genes represented on the arrays, they identified 20 as dynamically expressed. Therefore, our data taken together with these three publications convincingly show that expression of the APPG genes is dynamic during larval development. The mechanism by which the episodic expression of APPG genes is regulated is not yet known but the larval timing gene *nhr-23* may be involved since expression of several APPG genes is dampened when animals are treated with nhr-23 RNAi (Kouns et al., 2011) (supplementary material Table S8).

Another prion-like protein, encoded by the gene *pqn-47*, is required for shedding of the cuticle during the molt (Russel et al., 2011) but diverges from the APPG proteins in amino acid sequence (supplementary material Fig. S1), is not differentially expressed during the molt (supplementary material Table S1), and shows a different expression pattern from APPG genes (Russel et al., 2011).

### Do APPG proteins form functional amyloid?

Our microarraay analysis reveals that although the animals are behaviorally quiescent, they are metabolically active, with approximately 10% of all genes being induced. Many of the induced genes may be involved in cuticular synthesis, since even after culling for genes associated with lethargus behavior or with cuticle escape, the cuticle gene set contains 873 induced genes. Most of the genes with the highest levels of induction are the APPG genes and the APPG proteins we tested appear to localize to the pharyngeal cuticle. PQN proteins were originally identified by *in silico* proteomic analysis looking for proteins encoded in the *C. elegans* genome that, based on a high abundance of the amino acids glutamine and asparagines, were predicted to form prions (Michelitsch and Weissman, 2000). APPG proteins contain high percentage (≥9%) of cysteines, which are in an ordered distribution (supplementary material Fig. S2). These clustered cysteines may facilitate inter- and/or intra-molecular cysteine bridges required for the construction of the pharyngeal cuticle, much like the role of regularly-spaced cysteines in body cuticle collagens (Johnstone, 1994).

In contrast to the *C. eleg*ans body cuticle, which is collagenous, chitin biosynthetic enzymes, and not collagens, are expressed in the *C. elegans* pharynx (Veronico et al., 2001). Chitin synthase 2 (CHS-2) is expressed in the pharynx, and treatment of animals with RNAi against CHS-2 results in misshaped grinders and larval arrest. The presence of chitin in the pharynx has been confirmed by the binding of a chitin specific fluorescent protein (Zhang et al., 2005).

We propose that the APPG proteins are components of an extra-cellular cuticular matrix that lines the buccal cavity, the pharyngeal cuticle, and makes up the grinder. These proteins may interact in a manner that has stoichiometric constraints. As in other organisms with chitinous cuticles, this protein matrix is embedded with chitin, enhancing its role as an environmental barrier (Willis, 2010). The pharyngeal cuticle not only plays an active role in bacteria transport and maceration but also functions as a barrier protecting the organism from both live bacteria and bacterial lysates. While we have provided functional evidence in the formation of these structures for two (*abu-14* and *abu-6*) of the 29 APPG genes, all 29 genes share striking molecular signatures at both the protein sequence and gene expression levels, suggesting that they have similar functions. While our consideration of APPG gene function is centered on pharyngeal cuticle formed during development, rare additional expression outside the pharynx (supplementary material Fig. S3 and http://nematode.lab.nig.ac.jp), suggests a subset may function elsewhere too.

We offer the first plausible function for the “prion-like” nature of these proteins (Michelitsch and Weissman, 2000). We have shown co-localization of an amyloid stain with APPG proteins, leading us to propose that APPG proteins themselves form amyloid. The pharyngeal cuticle not only plays an active role in bacteria transport and maceration but also functions as a barrier protecting the organism from both live bacteria and bacterial lysates, which contain proteases. Functional amyloid has chemical and physical properties that would be beneficial for such a task.

### APPG proteins and innate immunity

APPG genes have been described as having a role in innate immunity, being required for the prevention of pathogenic infection (Haskins et al., 2008; Sahu et al., 2012). Reduction of APPG gene function accelerates the lethal effects of exposure to Vibrio cholera cytolysin (Sahu et al., 2012). Given that an organism's exoskeleton is a barrier to the pathogens and toxins in the environment, experimental paradigms that disrupt this structure would be expected to result in increased susceptibility to infection. Indeed, *phm-2* mutants, which have a defective pharyngeal cuticle (Avery, 1993), are hypersensitive to infection (Labrousse et al., 2000; Kim et al., 2002; Smith et al., 2002).

### Endoplasmic reticulum stress and larval molts

Members of the APPG were identified based on their induction by endoplasmic reticulum (ER) stress in mutant animals unable to mount a canonical ER stress response. We therefore considered the possibility that the APPG proteins may be induced in reaction to changes in protein homeostasis imposed by molting, a phenomenon which could tax the animal's protein assembly machinery. Although we detected no canonical ER protein chaperones (e.g. *hsp-3* and *hsp-4*) as induced during L4 lethargus or other cuticular synthesis periods (supplementary material Table S1), we considered the possibility that the APPG proteins are functioning as non-canonical chaperones for proteins being generated for the worm's exoskeleton. We therefore recreated the ER stressing conditions originally used to define the *abu* genes, with the difference that we took care to harvest only young adults and avoiding molting animals. We did not observe induction of the four APPG genes detected by our qPCR probe, suggesting that either (1) competence for APPG gene induction by ER stress is limited to the L4 stage, or (2) the previously-used experimental paradigms resulted in enrichment for molting animals. It would be difficult to test the former explanation since L4 animals, both stressed and unstressed, would have to be harvested at the precise same point in the L4 stage while staying fully clear of the molt. Instead, we performed this experiment in L1 animals and saw no APPG gene induction with ER stress. We favor the latter explanation, that inclusion of molting animals explains prior results in the literature. This explanation is supported by the recent analysis by Snoek et al., who suggest that a high percentage (27%) of prior gene expression studies are unintentionally influenced by variation in larval developmental timing between samples (Snoek et al., 2014). Nevertheless, since in our study we tested only four genes, caution must be exercised when extending this explanation to all 29 APPG genes.

We propose that some or all of the 29 APPG proteins are used in building the pharyngeal cuticle and grinder. The burden of expressing such high levels of protein during a discrete window of time, in an organ that generally functions as an excitable, contractile tissue does indeed raise the possibility that protein homeostasis is challenged by this event. In addition, APPG proteins are composed of long stretches of intrinsically-disordered domains, which may require “nanny” proteins to protect them from degradation (Tsvetkov et al., 2009). The implication is that the APPG proteins may themselves be clients for hitherto undiscovered chaperone proteins, analogous to the mechanism used by *Escherichia coli* to create amyloid biofilm (Evans and Chapman, 2014). Finally, targeting of the proteins to precise locations on the apical side of pharyngeal myoepithelial cells would require dedicated cellular machinery, such as for example the recently described RAB-6.2 and its regulator EAT-17 (Straud et al., 2013).

## MATERIALS AND METHODS

### General methods

*C. elegans* was cultured by standard methods (Stiernagle, 2006) on the surface of NGM 1.5% agar Petri dishes of 5.5 cm diameter. Animals were fed the *E. coli* strain DA837 (Davis et al., 1995) and cultivated at 20°C unless noted otherwise. The wild-type strain used was N2 (Brenner, 1974), which was obtained from the Caenorhabditis Genetics Center (CGC) in 2007 and was freshly thawed every 6 months. Other strains obtained from the CGC include: EG4322 *ttTi5605 II; unc-119(ed3)* III, SJ17 *xbp-1(zc12)* III; *zcIs4[hsp-4>gfp]*, VC1330 *abu-14(ok1789)/mIn1[mIs14 dpy-10(e128)] II*. Strains used in this study are listed in supplementary material Table S10.

### Back-crossing *abu-14(ok1789)*

Non-green males from a cross between N2 males and *abu-14(ok1789)/mIn1* heterozygotes were crossed back to *mIn1* homozygotes to rebalance the strain. The presence of the *abu-14* chromosome was detected based on the segregation of arrested larva. After five back-crosses, the presence of the *ok1789* deletion in genomic DNA was confirmed by PCR amplification using oligonucleotides oNQ1018 and oNQ1019 (supplementary material Table S11) followed by gel electrophoresis.

### RNA isolation, cDNA synthesis, pre-amplification, and microarray hybridization

Embryos were collected by the alkaline bleach method (Stiernagle, 2006) from populations of N2 hermaphrodite animals on the first day that the bacteria on the agar plate were depleted. The collected embryos were kept in M9 buffer without bacteria for 16 hours to permit hatching and developmental arrest at the L1 diapause stage. Developmentally arrested L1 animals were then transferred at a density of 3 animals per microliter in a 100 µL volume onto NGM plates fully covered with DA837 bacteria and allowed to develop at 20°C. Staged animals were collected in batches of 20 animals into 50 µL of ice-cold Trizol reagent (Invitrogen) in a 1.5 mL microfuge tube, vortexed for 30 seconds, and then flash-frozen by immersion in dry ice in ethanol. Staged animals were collected by hand at room temperature (22.5°C) under 50× (total magnification) stereomicroscopic (Zeiss Stemi 2000) visual guidance using a sterile platinum wire coated with bacteria.

Late fourth larval stage (L4) animals were identified based on age (42 hours after feeding L1 arrested animals), size, and a mid body ventral clearance with a dark shadow in its center, which correspond to the developing reproductive structures. L4 animals were all moving and feeding. L4 lethargus animals were identified 45–50 hours after feeding arrested L1s based on quiescence of locomotion and pharyngeal pumping. By checking the cohorts of animals every 10 minutes and removing animals in lethargus, we ensured that any newly-identified lethargus animal was within 10 minutes of the start of L4 lethargus. These early L4 lethargus animals were transferred to a fresh NGM plate covered with a lawn of DA837 bacteria. A subset of these L4 lethargus animals were allowed to age for 30 minutes at room temperature before collection. Thus, the L4 lethargus animals were collected 30–40 minutes after cessation of pharyngeal pumping. Prior to collecting each animal, it was verified by visual inspection of its behavior to still be in lethargus. Another subset of these L4 lethargus animals were allowed to age for six hours at room temperature before collection during the early adult stage. These early adult animals, which were at an age of approximately four hours after the L4-adult molt, had not yet begun making embryos. Third larval stage (L3) animals were collected based on age (32 hours after feeding L1s) and active behavior. L3 lethargus animals were collected based on age (37 hours after feeding L1s) and quiescence of locomotion and feeding.

Following collection in cohorts of 20 animals per tube, samples were pooled such that each sample of L4, L4 lethargus, and young adult animals had a total of 100 animals (5 tubes per pooled sample), and each sample of L3 and L3 lethargus animals had a total of 200 animals (10 tubes per pooled sample). A total of six samples, each with 100 animals, were collected for L4, L4 lethargus, and Adult stages, and a total of four samples, each with 200 animals, were collected for L3 and L3 lethargus stages. To extract the RNA, 40 µL chloroform was added to 200 µL of Trizol worm suspension, and then centrifuged at 13,000 RPM in an Eppendorf 5415C centrifuge for 15 minutes at 4°C. The top aqueous phase was then transferred to a fresh microfuge tube, combined with equal volume of chilled 70% ethanol, and mixed. The RNA was then isolated using RNeasy MinElute columns (Qiagen) according to manufacturer's instructions, eluted in 12 µL of RNAse-free water and stored at −80°C until further processing.

Experiments to assess the quality of the isolated RNA, cDNA synthesis, pre-amplification, and microarray hybridization were all performed at the University of Pennsylvania Molecular Profiling Facility. The A_260_/A_280_ ratio ranged 1.56–2.17 (average 1.95, standard deviation 0.16), and the A_260_/A_230_ ratio ranged 0.27–1.77 (average 0.97, standard deviation 0.43). The average ± SD total RNA in nanograms isolated per pooled sample was 22.3±7.0 (L4), 18.6±3.5 (L4 lethargus), 75.3±10.3 (young adults), 29.1±15.9 (L3) and 19.7±3.5 (L3 lethargus). The quality of RNA was further assessed using Agilent 2100 Bioanalyzer. Based on this quality assurance analysis, one sample each from the L4, L4 lethargus, and Adult groups was eliminated from further processing. cDNA synthesis and preamplification were performed using Nugen Ovation RNA Amplification System V2 (Catalog no. 3100-60). The amplified cDNA was then fragmented and labeled for hybridization to Affymetrix GeneChip *C. elegans* Genome Array (Catalog no. 900384).

### Quantitative reverse transcriptase polymerase chain reaction (qRT-PCR) analysis

Developmentally-arrested L1 larvae were prepared by the alkaline bleach method and plated at a density of 300 animals per plate on NGM agar fully seeded with DA837 bacteria in Petri dishes of 5.5 cm diameter. After 48 hours, worms in L4 lethargus were identified by their reduced movement and absent feeding and transferred to a new seeded NGM plate. After two to three hours, animals that had completed L4 lethargus as assessed by the resumption of feeding and moving were transferred to experimental plates. In the Tunicamycin experiment, these young adult animals were transferred to NGM agar that had been supplemented with Tunicamycin dissolved in DMSO to a final concentration of 5 µg/mL (The final DMSO concentration in the agar was 0.5%.) or to control NGM plates containing 0.5% DMSO. (Efficacy of the Tunicamycin in the induction of an unfolded protein response was first established by showing increased fluorescence in the strain SJ4005, which contains an integrated transgene of GFP under the control of the *hsp-4* promoter.) The animals were exposed to this treatment for five hours at 20°C, and then collected for qRT-PCR analysis. For exposure to the pathogenic *Pseudomonas* strain PA14, young adult animals, which were within 1 hour of completing L4 lethargus, were transferred from plates fully-seeded with DA837 bacteria to plates fully-seeded with PA14 bacteria. The control animals were maintained on DA837 bacteria. After 4 hours of cultivation at 20°C, the animals were collected for qRT-PCR analysis. For L4 lethargus versus young adult *abu* gene expression analysis, worms in L4 lethargus were either harvested directly for qRT-PCR or were transferred to another seeded NGM plate and allowed to age for 4–6 hours before harvesting as young adults for qRT-PCR analysis.

Fifty worms per biological replicate were snap frozen in Qiagen RLT Lysis buffer. Lysis and homogenization were achieved with the addition of 5 mm Zirconium oxide beads, and then subjecting the worm and bead mixtures to milling in Bullet Blaster (NextAdvance). Eluted RNA was immediately reverse transcribed to cDNA using random primers and SuperScriptIII® (Invitrogen). qPCR reactions were performed using Taqman® Gene Expression Mastermix on a Applied Biosystems 7500 platform. Primers and probes are listed in supplementary material Table S11. Because of the high degree of nucleotide sequence identity among the genes *abu-6*, *abu-7*, *abu-8*, and *abu-15*, we chose primers that detected all four genes. For each condition, 4 biological samples were collected and for each biological sample, 2 technical replicates were performed. The reference gene used was *cdc-42*, which has been demonstrated to show low variability in expression between experiments (Hoogewijs et al., 2008).

### Transgenics and transgene nomenclature

Transgenic animals were made by microinjection of DNA by standard methods (Mello and Fire, 1995). Constructs, selectable markers, concentrations injected, and number of transgenic lines analyzed are listed in supplementary material Table S9. We denote promoters by placing a “>” after the promoter of the gene and protein fusions by placing “::” between the parts of the protein. For example, we denote a fusion protein containing ABU-14 and GFP and expressed under the control of the *abu-14* promoter as “*abu-14>abu-14::GFP*”. Animals expressing the *abu-14>abu-14::GFP* transgene were fed the *E. coli* strain HB101.

### Microscopy and video

Animals were prepared for observation by immobilization in a 5 µL drop of 15 mM levamisole on a 4% agar pad. Worms were observed under 40×, 63×, and 100× objective lens using a Leica DM5500B compound microscope equipped with differential interference contrast optics and epifluorescence. Images were captured using a Hamamatsu C4742-95 digital camera controlled with Leica LAS © software.

To make a video of grinder turnover during L4 lethargus, we immobilized the animals against agarose pads using polystyrene nanoparticles (Kim, E., et al., 2013). Images were captured at 3.75 frames per second using an Imaging Source DMK 31BU03 camera on a Leica DM 2500 P upright microscope at 63× and analyzed using IC Capture 2.2 software (Imaging Source). To make the video, one out of every 36 images was used and played at 30 frames per second.

### Grinder width measurements in developing larvae

Animals were washed off a cultivation plate that was nearly depleted of food in order to leave mostly unhatched eggs on the plate. The eggs were transferred to a fresh NGM plates seeded with bacteria, taken care to avoid young larvae. Every 15 minutes, newly-hatched animals were transferred to a fresh seeded NGM plate. Hence, the birth time of these animals was known with a 15-minute precision. The animals were aged in a 20°C incubator. Individual animals were imaged on agar pads using the differential interference contrast (DIC) optics on a Leica DM5500B upright microscope equipped with a 100× Plan Apo oil-immersion objective lens with N.A. = 1.4. After image acquisition, the width of the grinder was measured by drawing a line across the refractile grinder using the quantification tools in LAS© software.

### Phenotypic analysis of *abu-6(RNAi)*, *abu-14 (ok1789)* mutants, and *abu-14* transgenics

Growth rate was measured by first synchronizing first larval stage worms using the hypochlorite bleach method, and then growing on agar fully seeded with DA837 bacteria. RNAi and control animals were plated at a density of 300 L1s per agar plate. 48 hours after re-feeding, the larval stage of the animals was identified based on the developmental stage of the reproductive system. *abu-14* mutant animals were assessed at 72 hours after re-feeding. The rare *abu-14* mutants that reached adulthood were sterile. While the growth retardation and abnormal grinder phenotypes of *abu-14* mutants were rescued with a genomic fragment containing the *abu-14* gene injected at a concentration of 2 mg/liter, the sterility phenotype was not rescued. We do not know whether the sterility is caused by a tightly linked mutation in another gene, or whether it is caused by the *abu-14(ok1789)* mutation yet was not rescued by the *abu-14* genomic DNA due to poor germline expression of transgenes, a common property of *C. elegans* transgenes (Kelly and Fire, 1998). The DNA construct encoding the ABU-14::GFP fusion protein was injected at a concentration of 0.2 mg/liter. One of two transgenic lines made by injecting this low DNA concentration rescued the growth defects and grinder abnormalities of *abu-14(ok1789)* mutants but did not show green fluorescence in the pharynx. An injection at much higher 50 mg/liter concentration of this construct caused the animals to grow extremely slowly but did show green fluorescence in the pharyngeal grinder. However, we found that after passaging the transgenic animals for several generations, the transgene was silenced and we could no longer detect fluorescence. We therefore remade the transgenic animal using superfolder GFP (sfGFP) (Pédelacq et al., 2006) fused to the ABU-14 c-terminus. We were able to generate transgenic animals with low (0.5 ng/µL) concentration of abu-14>abu-14::sfGFP, and these animals showed bright green fluorescence localized to the pharyngeal cuticle.

Analysis of feeding was performed by cultivating animals on GFP-labeled *E. coli* bacteria. Two days after the L4 stage, animals were inspected with a Leica DM5500B compound fluorescent microscope at 63× magnification. We counted the fraction of animals whose pharyngeal corpus was stuffed with bacteria and the fraction of animals that contained intact bacteria in the anterior intestine.

### Staining with Congo Red

To prepare Congo Red NGM agar plates, 100 µL of a saturated solution of Congo Red in distilled water was added to the agar surface of NGM plates. After 1–2 days of dye equilibration into the agar, the plates were seeded with bacteria. Animals were placed on the surface of the Congo Red plates for one hour. The animals were then transferred to regular NGM plates and allowed to destain for 1–5 hours prior to analysis by epifluorescence.

### Analysis of microarray data

Probe intensity data from all Affymetrix arrays were read into the R software environment (http://www.R-project.org) directly from .cel files using the R/affy package (Gautier et al., 2004). Data quality was assessed using intensity histograms, box plots, scatter plots and principal components analysis. Normalization was performed using the robust multiarray average (RMA) method to form one expression measure per gene on each array (Irizarry et al., 2003). Probe sets were mapped to genes using BioMart (http://www.biomart.org, accessed 04-23-2010, http://www.wormbase.org release 195). Affymetrix control probe sets and probe sets not mapped to any genes were filtered from the data set before statistical analyses were conducted.

Analysis of variance (ANOVA) methods were used to determine differential gene expression using the R/maanova package (Wu et al., 2003). Models were fit separately for the comparisons: [1] L4 Lethargus versus L4, [2] L4 Lethargus versus Adult, [3] L3 Lethargus versus L3, [4] embryo (−2 hrs) versus L1 from a previously published data set (Baugh et al., 2009) and [5] L1 Lethargus versus L1 from Baugh et al. (Baugh et al., 2009). Specifically, the model Y_i_ = μ+STATE+ε_i_ was used to fit gene expression measures, where μ is the overall mean for probe set *i*, STATE is the effect for developmental state, and ε_i_ captures random error. Statistical tests were conducted for each model fit where the null hypotheses were H_0_: L4 Lethargus  =  L4, H_0_: L4 Lethargus  =  Adult, H_0_: L3 Lethargus  =  L3, H_0_: embryo  =  L1, and H_0_: L1 Lethargus  =  L1, respectively. All statistical tests were performed using Fs, a modified F-statistic incorporating shrinkage variance components (Cui et al., 2005). P-values were calculated by permuting model residuals 1000 times. The p-values from comparisons to L4 Lethargus were multiplied by 2 so that differentially expressed transcripts were required to meet the significance criterion in both statistical tests in this case. All p-values were further corrected using the p.adjust() function in R using method  =  “BH” (Benjamini and Hochberg, 1995). For the L4L gene set (Fig. 1A), differentially expressed transcripts had a corrected p-value less than 0.05 and were expressed in the same direction relative to Lethargus in both comparisons (L4 Lethargus vs. L4 and L4 Lethargus vs. Adult). The CUT gene set (Fig. 1A) was determined using a less stringent threshold of 0.30 between conditions from the Baugh et al. data set (Baugh et al., 2009) and expression in the same direction as the L4L data set. Similarly, to identify gene differentially expressed between L1 lethargus (15 hour time point from Baugh et al., 2009) and L1 (6 hour time point from Baugh et al., 2009) and between L3 lethargus and L3, the false discovery rate threshold was set to 0.3.

### Comparing L4 lethargus microarray results to other gene expression studies

Gene expression results obtained here were summarized into a list of unique genes and compared to gene lists described in the literature using the fisher.test() function in R. Our gene expression results were reduced to two gene lists [1] up-regulated versus Lethargus and [2] down-regulated versus Lethargus using the following strategy. First, our differentially expressed transcripts were divided into lists of up- and down-regulated transcripts compared to Lethargus. For each of these lists, the probe set with the largest F-statistic was selected to represent the gene. A changed gene was further constrained to have at least a 2-fold change compared to Lethargus in each comparison (L4 Lethargus versus L4 and L4 Lethargus versus Adult). The gene universe was taken to be the list of unique genes after probe set filtering (described above). A two-by-two contingency table assessed overlap between genes in our study (i.e., changed in one direction or not changed in that direction) and selected gene lists from the literature (i.e., changed in one direction or not changed in that direction in the study).

### Accession numbers

Microarray data are available for download from NCBI Gene Expression Omnibus (accession GSE46291) at http://www.ncbi.nlm.nih.gov/geo/query/acc.cgi?acc = GSE46291.

## Supplementary Material

Supplementary Material
