# Supplementary material for: Dynamically-expressed prion-like proteins form a cuticle in the pharynx of Caenorhabditis elegans
Source: Biol Open. 2014 Oct 31;3(11):1139–49. doi: 10.1242/bio.20147500 (PMC4232772; doi:10.1242/bio.20147500)
Supplement: Supplementary Material [file supp_3_11_1139__index.html]

Dynamically-expressed prion-like proteins form a cuticle in the pharynx of Caenorhabditis elegans — Dynamically-expressed prion-like proteins form a cuticle in the pharynx of Caenorhabditis elegans — Supplementary Material 

# Dynamically-expressed prion-like proteins form a cuticle in the pharynx of *Caenorhabditis elegans*

## bio.20147500 Supplementary Material

**Files in this Data Supplement:**

- Supplementary Material - Julia B. George-Raizen et al. doi: 10.1242/bio.20147500
- Movie 1 - **The pharyngeal grinder grows during the molt.** Time lapse video recording of the grinder during the L4 to adult molt. The recording is played 300 times faster than real time.
- Figure S2 - **ABU/PQN paralog group protein sequences, all with at least 9% cysteines (highlighted in yellow) and with at least one CxxxCxxxC PFAM domain.** The predicted signal sequences, as detected by the SignalP v4.0 program (Petersen et al., 2011), are underlined.
- Table S1 - **L4L UP gene set.** Gene transcripts in this set are up-regulated at 5% FDR between L4 lethargus and L4 AND between L4 lethargus and 4-hour old adults. FC denotes Fold Change and FDR denotes False Discovery Rate.
- Table S2 - **L4L DOWN gene set.** Gene transcripts in this set are down-regulated at 5% FDR between L4 lethargus and L4 AND between L4 lethargus and 4-hour old adults.
- Table S3 - **CUT UP gene set.** Gene transcripts in this set are in the L4L UP gene set AND are also up-regulated at 30% FDR between 2-hour old embryos and 3-hour old L1 animals.
- Table S4 - **CUT DOWN gene set.** Genes in this gene set are in the L4L DOWN gene set AND are also down-regulated at 30% FDR between 2-hour old embryos and 3-hour old L1 animals.
- Table S5 - **Gene expression of abu/pqn paralog group genes during cuticular synthesis stages.** Shown are the fold changes between four cuticular synthesis stages and the contiguous larval stage. The left two columns show analysis of data collected in this study and the right two columns show analysis of data collected by Baugh et al. (Baugh et al., 2009). DE denotes Differentially-Expressed.
- Table S6 - **Predicted targets of abu-6 (RNAi).** Targets are predicted using http://dscheck.rnai.jp (Naito et al., 2005). mis=0 denotes number of perfect 19-nt matches within the target gene to the conceptually diced double stranded RNA sequence. mis=1 denotes number of matches to the diced double stranded RNA sequence with 1 mismatch and mis=2 denotes the number of matches to the diced doubles stranded RNA sequence with 2 mismatches.
- Table S7 - **Gene expression changes observed in this study and in published studies.** Shown are the fold changes determined by microarray analysis of genes in the abu/pqn paralog group as well as selected other genes.
- Table S8 - **Statistical comparison with published data sets.**
- Table S9 - **Transgenes used in this study.** All constructs were made by PCR method of Hobert (Hobert, 2002). DNA constructs encoding fusions between APPG genes and GFP were gel purified prior to injecting.
- Table S10 - **Strains used in this study.**
- Table S11 - **Oligonucleotides used in study.**
