# Supplementary material for: Dynamically-expressed prion-like proteins form a cuticle in the pharynx of Caenorhabditis elegans
Source: Biol Open. 2014 Oct 31;3(11):1139–49. doi: 10.1242/bio.20147500 (PMC4232772; doi:10.1242/bio.20147500)
Supplement: Supplementary Material [file supp_bio.20147500_bio.20147500-s1.pdf]

 Biology Open (2014) 000, 1–11 doi:10.1242/bio.20147500

Supplementary Material

Julia B. George-Raizen et al. doi: 10.1242/bio.20147500

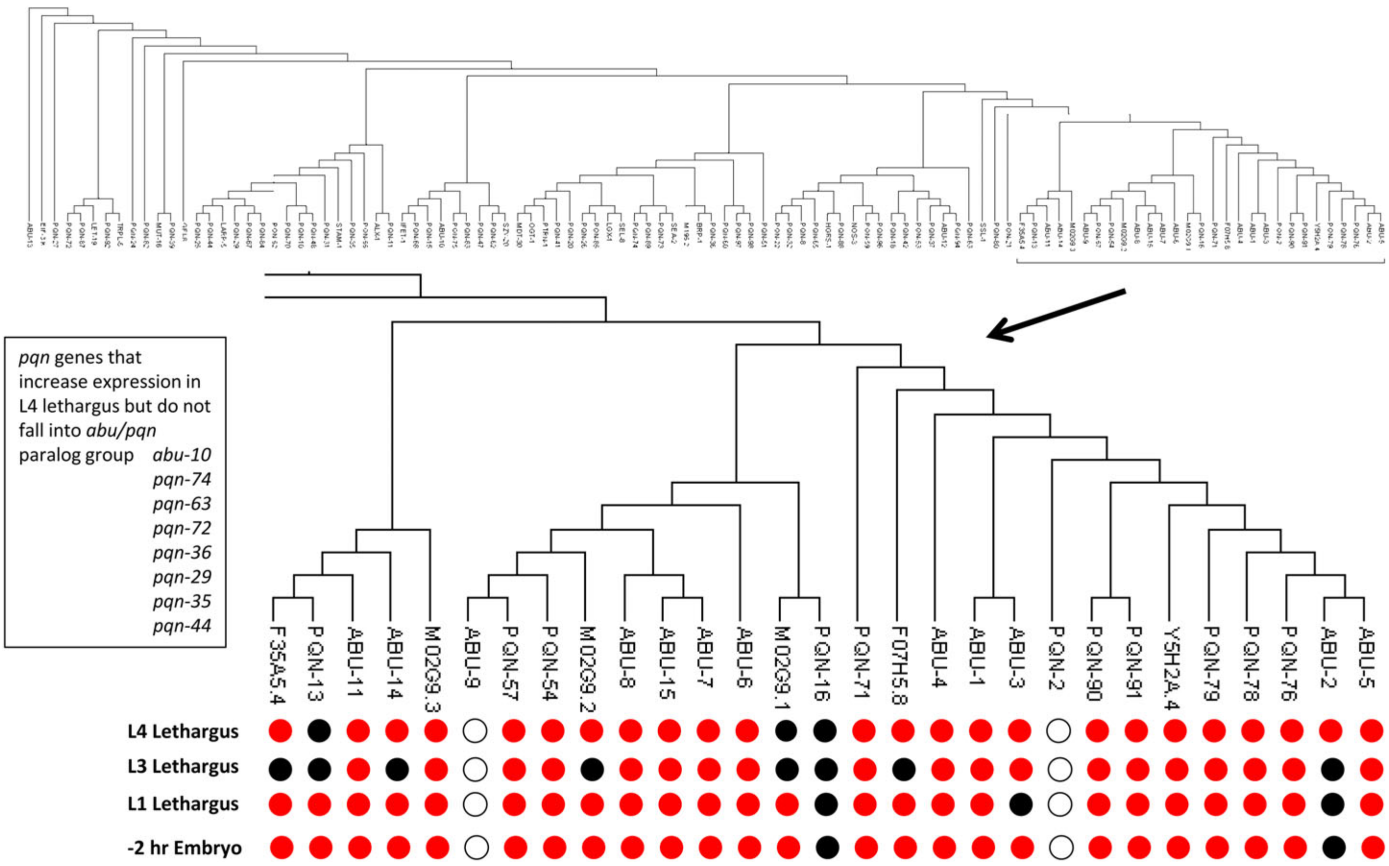

Fig. S1. Phylogenetic analysis of the 106 *C. elegans* PQN proteins showing clustering of 29 ABU/PQN proteins. Red circles denote up-regulated in stage, black circles denote not up-regulated in stage, and open circles denote not represented on the microarray. Inset on left lists *pqn* genes that increase expression in L4 lethargus but do not fall into the *abu/pqn* paralog group. The evolutionary history was inferred by using the Maximum Likelihood method based on the JTT matrix-based model (Jones et al., 1992). The tree with the highest log likelihood (−6435.3800) is shown. Initial tree(s) for the heuristic search were obtained automatically by applying Neighbor-Join and BioNJ algorithms to a matrix of pairwise distances estimated using a JTT model, and then selecting the topology with superior log likelihood value. The tree is drawn to scale, with branch lengths measured in the number of substitutions per site. The analysis involved 106 amino acid sequences. All positions containing gaps and missing data were eliminated. There were a total of 28 positions in the final dataset. Evolutionary analyses were conducted in MEGA5 (Tamura et al., 2011).

Fig. S2. See supplementary webpage.

Biology Open (2014) 000, 1–11 doi:10.1242/bio.20147500

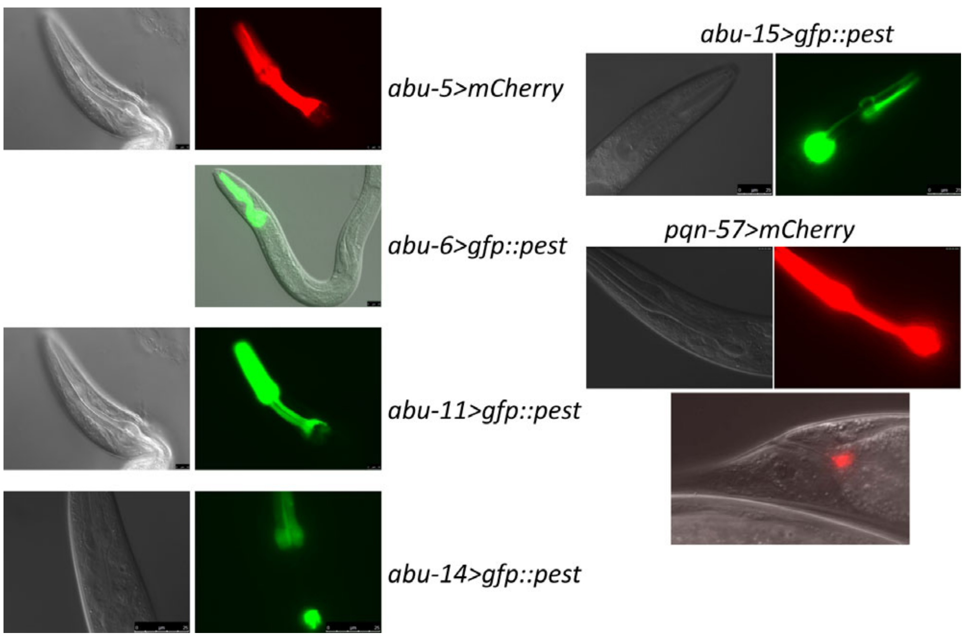

Fig. S3. Expression analysis of *abu/pqn* paralog group genes using fluorescent reporters fused to the promoter of the gene. In every case, expression is in pharyngeal muscle. *pqn-57* was also expressed in a cell at the origin of the rectum.

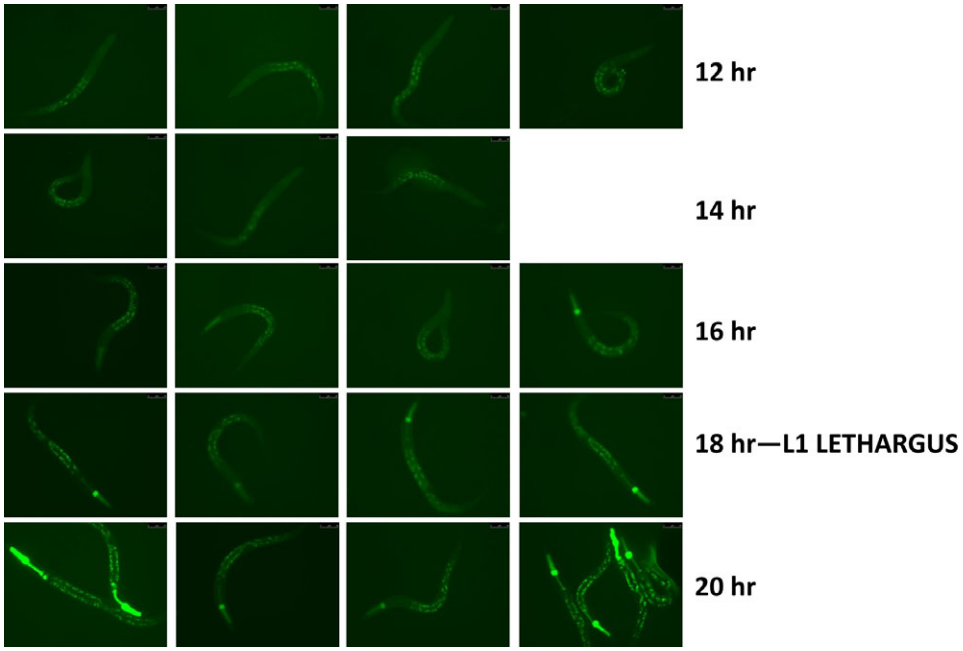

Fig. S4. Dynamic larval expression of the APPG gene *abu-11*. Shown are fluorescent images of *abu-11*>*gfp* transgenic animals at the indicated time after feeding of L1-arrested animals. Fluorescent intensity increases approximately 2 hours after lethargus. L1 lethargus is approximately two hours delayed relative to wild-type animals because the strain carries an *unc-119* mutation, which is not rescued in every cell. Images were captured using the same camera exposure time.

Biology Open (2014) 000, 1–11 doi:10.1242/bio.20147500

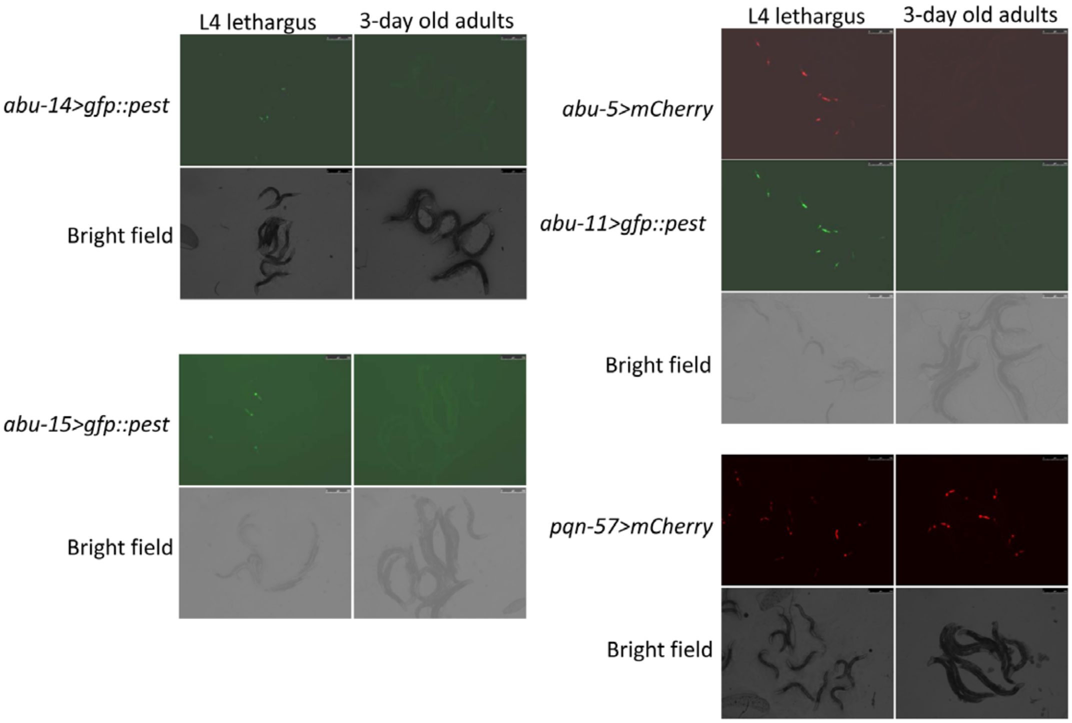

Fig. S5. Reduced expression of APPG genes in adult animals. In every case except one, expression is much weaker in three-day old adults than in L4 lethargus animals. *pqn-57* pharyngeal expression appears to persist in adults. Microscope and camera settings were identical within each genotype.

Tables S1–S11. See supplementary webpage.

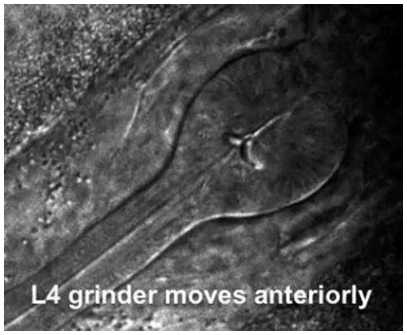

Movie S1. The pharyngeal grinder grows during the molt. Time lapse video recording of the grinder during the L4 to adult molt. The recording is played 300 times faster than real time.

Biology Open
