# Supplementary material for: Dynamically-expressed prion-like proteins form a cuticle in the pharynx of Caenorhabditis elegans
Source: Biol Open. 2014 Oct 31;3(11):1139–49. doi: 10.1242/bio.20147500 (PMC4232772; doi:10.1242/bio.20147500)
Supplement: Supplementary Material [file supp_bio.20147500_FIGURE_S2.docx]

**ABU-5**

MRFIAIAALLASSLLLVEGTSIRDKRQSCGCAPKVQPSCSCQRTTYTQPQQYSCSCQNTAPVQTSCSCAQPVQQLTYQVQATQ

CAPACQQSCQRQCQSAPSVSN

CQSSCQQTCQTSSCYTPVAPAPVQ

CQPSCMPVCEQSCVAPAPQVISLNLEVVPQ

CQQQCAPQCQQPSAPQ

CQQCQNTCQQYAPV

CQQQCAPQCTFPSAPA

CQQCQTSCQQTQQ

CQQQCIPQCQQPAAPQ

CQQCQSACQSPVVAPQIVTVILEPSVSQSAQ

CVPQCQQSCQQQCIQQQQPMQQ

CAPACTQSCYQSCSTAQPVQMPCLTQSVNSCSCQQNYSPCGNGQCCMRK

**ABU-2**

MRFIAIAALLASSLLLIETTSIREKRQSCGCAPKVQPSCSCQQTPYTQPQETSCSCQNTAPVQTSCSCAQPVQQQTYLVPTSQ

CAPACQQSCQIHCHSAPFVQE

CQSSCQQSCQTTSCYTPPAPAQ

CMPQCQQQCSQQCVQTQPVQH

CQSQCHQQCVQRCAPIVAPQLININLEAPAQ

CSQCQQSCQQQCVQQQYSVQQ

CNQHCNLECQPTCQQAVSQ

CQQQCAPQCQQPSAPQ

CQQCQSSCQQTQQ

CQQQCIPLCNQPSAPA

CQQCQSACQSPVVAPQVVNVMLDVSVAQSAQ

CQPQCQQSCQQQCIKQQYPVQQ

CIQPCTVECQTACQQAVPQ

CQQQCAPQCYQPSAPQ

CQQCQNTCQQFAPV

CQQQCASQCLQPAAPQ

CQQCQNTCQQFAPV

CEQQCASLCHQPSAPQ

CQQCQNTCQQFAPV

CEQQCAPQCYQPSAPQ

CQQCQASCQQTQQ

CQQQCIPQCQQPSAPQ

CQQCQSACQFPVVAPQVVAVILEASVSQSAQ

CEPQCQQSCQQQCIQQLQPIQQ

CATACTQSCSQSCSAAQPAQMPCITQSANSCSCQQNYSPCGNGQCCKRR

**PQN-76**

MRFIAIAALLASSLLLVEGTSIRDKRQSCGCAPKIQPSCSCQRTTYTQPQQYSCSCQNTAPVQTSCSCAQPVQQLTYQVQATQ

CAPACQQSCQRQCQSAPSVSN

CQSSCQQTCQTSSCYTPVAPAPVQ

CQPSCMPACEQSCVAPAPQVISLNLEVVPQ

CQQQCAPQCQQPSAPQ

CQQCQNTCQQYAPV

CQQQCTPQCTLPSAPA

CQQCQTSCQQTQQ

CQQQCIPQCQQPAAPQ

CQQCQSACQSPVVAPQIVTVILEPSVSQSAQ

CVPQCQQSCQQQCIQQQQPMQQ

CAPACTQSCYQSCSTAQPVQMPCLTQSVNSCSCQQNYSPCGNGQCCMRK

**PQN-78**

MRFIAIAAILASSLLLVEGTSIRDKRQSCGCAPKVQPSCSCQRTTYTQPQQYSCSCQNTAPVQTSCSCAQPVQQLTYQVQATQ

CAPACQQSCQRQCQSAPSVSN

CQSSCQQTCQTSSCYTPVAPAPVQ

CQPSCMPVCEQSCVAPAPQVISLNLEVVPQ

CQQQCAPQCQQPSAPQ

CQQCQNTCQQYAPV

CQQQCAPQCTLPSAPA

CQQCQTSCQQTQQ

CQQQCIPQCQQPAAPQ

CQQCQSACQSPVVAPQIVTVILEPSVSQSAQ

CVPQCQQSCQQQCIQQQQPMQQ

CAPACTQSCYQSCSTAQPVQMPCLTQSVNSCSCQQNYSPCGNGQCCMRK

**PQN-79**

MRFIAIAALLASSLLLVEGTSIRDKRQSCGCAPKIQPSCSCQRTTYTQPQQYSCSCQNTAPVQTSCSCAQPVQQLTYQVQATQ

CAPACQQSCQRQCQSAPSVSN

CQSSCQQTCQTSSCYTPVAPAPVQ

CQPSCMPACEQSCVAPAPQVISLNLEVVPQ

CQQQCAPQCQQPSAPQ

CQQCQNTCQQYAPV

CQQQCTPQCTLPSAPA

CQQCQTSCQQTQQ

CQQQCIPQCQQPAAPQ

CQQCQSACQSPVVAPQIVTVILEPSVSQSAQ

CVPQCQQSCQQQCIQQQQPMQQ

CAPACTQSCYQSCSTAQPVQMPCLTQSVNSCSCQQNYSPCGNGQCCMRK

**Y5H2A.4**

MLNVQNLVSWTISSVSLCPLGYDLLYVHLLTTLFHFQATSIRDKRQSCGCAPRVQPSCSCQRTTYTTQPQQYSCSCQNTA

PVQASCSCAQPVQHQSYQVQTSQ

CAPACQQSCQMQCQSAPFVNE

CQSQCQQSCQTSSCYAPAAPAPVQ

CMPQCQQQCTQQCVQTQPIQQ

CQPQCQQQCVQQCAPRVAPQIVSMNLEVPAQ

CPQCQQTCHRQCVQQQFPVQQ

CTQQCTLECQPTCQQAVPQ

CQQQCAPQCQQPSAPQ

CQQCQSACQSPVVAPQIITLEVVPQ

CQQQCAPQCQQPAAPQ

CQQCQTTCQQFAPV

CQQQCAPQCTLPSAPV

CQQCQTSCQQFAPV

CQEQCAPQCQQPAAPQ

CQQCQSACQSPVIAPQIITLEVVPE

CQQQCAPQCQQPSAPQ

CQQCQNTCQQFAPV

CQQQCAPQCTLPSAPA

CQQCQTSCQQTQQ

CHQQCIPQCQQPAAPQ

CQQCQSACQSPVVAPQIVTVILEASVSQSAQ

CEPECQQSCQQQCVQQQQPMIQ

CAPACTQSCSQSCSIAQPAQMPCMTESINSCSCQQNYSPCGNGQCCKR

**PQN-90**

MRFITIAAVLLVSSLLIVEASSIRDKRQSCGCASQVQPSCSCQQATQPQQYSCSCQNTAPVQTSCSCAQPVQQVTYQVQTSQ

CAPACQQSCQNQCQFAPSMNE

CQSSCQQTCQTSSCYTQIAAPAPVQ

CQPSCMPACEQSCVAPAPVTVQ

CVPQCQQKCTQQCVQAQPIQQ

CQPQCQQQCVQQCAPTVAPQVINVNLGISAQ

CAPQCQQQCQQQCLLQQVPVQQ

CYQECTQPCQTTCQQAAPQ

CVQQCAPQCQQPSAPQ

CQQCQNTCQQYAPV

CQQQCAPQCTLPSAPA

CQQCQTSCQQTQQ

CQQQCIPQCQQPAAPQ

CQQCQSACQSPVVAPQIVTVILEPSVSQSAQ

CVPQCQQSCQQQCIQQQQPMQQ

CAPACTQSCYQSCSTAQLVQMPCLTQSVNSCSCQQNYSPCGNGQCCKRM

**PQN-91**

MRFIVFVALFASSCLLIEATSIRDKRQSCGCASQVQPSCSCQQATQPQQYSCSCQNTAPVQTSCSCAQPVQQVTYQVQTSQ

CAPACQQSCQNQCQFAPSMNE

CQSSCQQTCQTSSCYTQIAAPAPVQ

CQPSCMPACEQSCVAPAPVTVQ

CVPQCQQKCTQQCVQAQPIQQ

CQPQCQQQCVQQCAPTVAPQVINVNLGISAQ

CAPQCQQQCQQQCLLQQVPVQQ

CYQECTQPCQTTCQQAAPQ

CVQQCAPQCQQPSAPQ

CQQCQNTCQQYAPV

CQQQCAPQCTLPSAPA

CQQCQTSCQQTQQ

CQQQCIPQCQQPAAPQ

CQQCQSACQSPVVAPQIVTVILEPSVSQSAQ

CVPQCQQSCQQQCIQQQQPMQQ

CAPACTQSCYQSCSTAQLVQMPCLTQSVNSCSCQQNYSPCGNGQCCKRM

**PQN-2**
MRFFAIAALLVSSCFLIEATTIRDKRQSCGCAPRVQPSCSCQRTTYTQPQQYSCSCQNTAPVQKSCSCAQPVQQQTYQIQASQ

CAPACQQSCQNQCQSAPSVSQ

CQSTCQQSCQTSSCYTPTTPAPVQ

CQPSCMPACEQSCVVQTPAAVQ

CVPQCQQQCQQQCVQTQPIQQ

CQPQCQQQCVQQCAPTTTAAPQIIKINMEISAQ

CVPQCQQSCQQQCVQQQVPAQQ

CNQQCTQQCQTTCQQAVPQ

CQQQCAPQCQQPSAPQ

CQQCQNTCQQAAPV

CQQQCAPQCQQQSAPA

CQQCQTSCQQTQQ

CQQQCTPQCQQPSAPQ
CQQCQSACQAPVATTAAPQAVTIILEASVSQSAQ

CEPQCQQSCQQQCVQQQQPMQQ

CAPACTQSCSQSCSAAQPAQMPCQTQSVNSCSCQQNYSPCGNGQCCKRK

**ABU-3**

MRFFAFAVFIVSSILLVE

CAPACQQSCQNQCKSAPFVNQ

CQSSCQQNCQTSSCSASATLAPVQHVPQ

CQQQCAPQCQQPAAPQ

CQQCQNTCQQSAPV

CQQQCAPQCQQQFAPA

CQQCQNSCQQTQQ

CQQQCTPQCQQPSTPQ

CQQCQSACQAPATAAPQISLNLEASVSQSAQ

CEPQCQQSCQQQCVQQQQSMQQ

CASACTKSCSQSCSQPAQMPCQTQTLNSCSCQQNYSPYGNGQCCKRK

**ABU-1**

MRFIAIAALIASSVLLAEATTIRDKRQSCGCAPRVQPSCSCQRTTYTQPQQYSCSCQNTAPVQKSCSCAQPVQQQTYQIQASQ

CAPACQQSCQNQCQSAPSVSQ

CQSTCQQSCQTSSCYTPTTPAPVQ

CQPSCMPACEQSCVVQTPAPVQ

CVPQCQQQCQQQCVQTQPIQQ

CQPQCQQQCVQQCAPTTTAAPQIIKINMEISAQ

CVPQCQQSCQQQCVQQQVPAQQ

CNQQCTQQCQTTCQQAVPQ

CQQQCAPQCQQPSAPQ

CQQCQNTCQQAAPV

CQQQCAPQCQQQSAPA

CQQCQTSCQQTQQ

CQQQCTPQCQQPSAPQ

CQQCQSACQAPVATTAAPQVVTIILEASVSQSAQ

CEPQCQQSCQQQCVQQQQPMQQ

CAPACTQSCSQSCSAAQPAQMPCQTQSVNSCSCQQNYSPCGNGQCCKRK

**ABU-4**

MRFIAIAALLASSFLLIEATATSTREKRQSCGCAPRVQPSCSCQQTTYAQPPQYSCSCQNTAPVQTSCSCAQPVQQQTYQVQASQ

CAPACQQSCQMQCQSAPSVSQ

CQSTCQQSCQASSCYTPAPVQ

CQPSCMPACKQSCVAPAPQIISLNLEVVPQ

CQQQCAPQCQQASAPQ

CQQCQNTCQQFAPV

CQQQCAPQCTISSAPQ

CQQCQTTCQQFAPV

CQQQCAPQCQQPSAPQ

CQQCQSACQSPVVAPVVAPQIVTVILEASVSQSAQ

CEPECQQSCQQQCVQQQQPMIQ

CAPACTQSCSQSCSIAQPAQMPCMTESINSCSCQQNYSPCGNGQCCKRK

**F07H5.8**

MTDVYKTVAEGSGAPKEHLPHCRRPAVPPRGVLRINDMKTNIILLLIVIGSYLVDSEQSLITRAKRQCPCAQLSQKSKCN

CYPIGSGMQRCKCSNEPQSSGCSCANKAQTSV

CQPSCQKSCVDSCVRNSHQPQLL

CDKTCQFTCNKACTPSTSTSSSSNFPPIKVVVNQQTKASQ

CPQACAQSCANSCTAQPNKSY

CIDMCIKSCQPTCVQAAVSINVKEFMRTSTTTLAPSRS

CVSACQPTCDAQCINVMKRYEVIIQKMPLAQK

CPSQCQPTCSPQCIQSVTVSIQTTAQPTTAS

CIPACQPACTPQCVQAVTTTET

CIPACQPACQPQCVERQLQIQIVTSQATQATRAPET

CIPACQPACQPQCVYQYLSSGSSGISTVPPVTKV

CISICQPACDPGCVAIYTTTPAPLH

CVSQCQPACLPSCTDTFTTTISLPKQ

CVPECQPACETKCIVAQIVLKIKNSYQQPQQLSQQYIPQSS

CVPQCQPACTQECVAAQPNFSVQINMIDETPSTSTSSPQ

CIPQCQPSCDQQCIQTYKIQVQQMNSQQKQQRQYN

CVPACQPTCEQSCIQSQYQVTIQQSYSKGNQGNS

CPSACQPACEPLCVQQITVQTTVPITKT

CVPQCQPACTQECVQQATTYTISIPMTTAAPS

CAPQCQPACDPQCISFTLKLPVMTTPPPTPS

CQPQCQPACQPSCMETYTFTIPMNDQSSKQ

CAPACQPVCDSKCIQNYQFEIVIPQADNN

CMPACTQSCQTSCVQQNSQSVPQ

CSTACTDSCRSSCVEIVKESAEPTFKLEIVLKKPMEETVT

CAPQCANQCVDQCKTQLLTQIEF

CLPACQNACQQNCPLQVEPCAMSGSQCNCSTGFSLCGNNQCCRKRRR

**PQN-71**

MRCVIILFAVALAVAQASSIRETRQASCGCAQSVQPTCSCQQASQQYSCSCQPSTPCSCASSQQYQLQTSQ

CMPACQQSCSQQCQSNTNTQ

CQPTCQQSCQTSSCNPMTSTPIPASQS

CLPECENRCMQQCTQQQTAQQ

CQPICQQQCQQECGSTGNMMYNNQDPYNQMQYGGYNQQGNQYQNQNQYQNPNQIQNQYNQNQYQNQNYYNPYQQTQ

CQQQCAPQCSQQTSTN

CQQCQNSCQNSNTQTITIYVQASPQTSQ

CVPQCQQQCQQQCQTRTTASQQ

CAPACSTSCNQSCNQPAQMACQPMQNSQCGCQQNYSPCGNVSGQCCLKK

**PQN-16**MKLYTHFILLVTFAVLASSSTIRDKRQNCKCSPPQSSCSCNSAIQSQTCTCHNTQQSTSASNCNCVLKSNSKSVPVTIKV
STKL

CAPACQQSCSQQCQDNQTIANNADTN

CISQCQAKCQARCGIQNGMGFQQSPATTTDAPIVIRLEITSGSSNSQ

CAPKCIQQCNNQCASQNQKTNQ

CANECNNQCANSCSPSSQTSSYSK

CVSQCAEQCSGSNTSNNQQ

CQQQCQSNTCGQYQSTVSTTTTTPIIQIVLNSSVLNSGSE

CEPQCENSCQNQCQAQQQSQQQ

CAQACQTGCVAQCQPANVS

CMPASTSTCQCQPNYSQCGNQCCRL

**M02G9.1, isoform b**
MGRSNSLRLLFLFCLLLALSTAASRRLKRQCGCSNFCNCQQQPVFFAQISLPACSCQQAPICQPQCPRAEINSD

CSATCVRACIPSCSKSTGNTFA

CSTTCESTCDKTCASAAQQAMSHIQVSPPNPQPLVPIAAAPTVDDS

CQNVCQNVCQGACVSQNSPPAV

CQQTCRQSCQFGCATNEQLPTTSSTSTNAPTIKITLNINDAYFDSN

CAPKCTQSCHSQCISQGNPAAS

CSNSCNTECSDKCSTRPVQAQVQQIQPQQVQITIPQT

CQSRCENRCLSTCTASQPSV

CAPSCSTACQLSCDSIGAATAPQSTNPTPVEVRAL

CTPTCMPQCLPSCTATTTTTTTQTPYIQTLAPAPAPAPVQIQIIQKQ

CVPSCMPACQPSCTNPVPLTTQAPVPVVNQ

CIPPCQPQCLQSCLEQHIQPQVVTQLPQ

CIPQCQPACEPQCIQETTTTTTTTTTQSPQVSTDEPVPDYPLDTTSTAPFP
STTEPEYEDEELTTIEPEVETTTESVELETEPEPEPEPEPEPDYTTPEPIDYTTVEPYDYPQYDQPSTPSTPSTPSPPST
STITYPTLPPSQPTLPPTQPQRDQ

CLPACQPTCNQNCIVAMQVLLYLS

CPEACRPACDPKCAYQYLRSNPSTPQHYNPTALASQSR

CVSACMPACLLDCVLPYFGDKTLPTLSPRVVVEQIPEEMPVDWGRLRDQATNQPLYLQASTQNPQGYLQTRPQA
YVQPASQSQPAKPSATTYVQPSSAQQSYVQPTQPSQQAHQTYQNAQPHPQAQTIHLQYKVTGAPSTPTLQFAQASQPTQS
FTLAQHQAQNQAKALHASHAAYNSPSQPVSGAYQPWSVPKTPLTMPGSLYNTASPVPGLGSQPKFQMQMVQVGGVPKNLN
PSTPVNYLTVNLKSAPGVQGSQAPIVKFKSPPTQISPAPPPAKQASRTIPLTRLFSTYGSVPKSLSNHIQSSSLATPQPS
ETNDVIDDNTPFVDVLPKNDPIEEE

CVPQCMPQCDPYCISQHRYSLRSPTASPGAS

CPKACMPECSNQCVATLQ

CPLSCQPACQPSCLQKPPIIKITVTGQQVG

CVEQCQPACDPKCIIATIKTPSQQPQFVVTTTQAPPAPAPQQQQQLAS

CPQLCQPQCTSQCVQQQQCPCQQTCQTGCQQHNPDARV

CQNVCVEVCASECPRTQPTVQQPAPQLVQQPIYQTVQAPLSYVPVVPVATSAPSQASGPQITINFAVPE

CIPVCEQSCNTQCVEKFPQEH

CGSVCNSQCQTACATQTPAVQAAPAPS

CQPQCQPACEPVCIAQQAQPVRIQINLATASSVLQASDA

CQPMCEQSCVQECQSTTLNVQAAT

CQPACQSICQQSCAPLGTSAPVMQTIPVVPVATAPVASTQL

CAPKCISDCQGLCKSNSPQ

CIQGCDASCQQLCGTAPTPAVPLTVNYN

CNLPCDQQCTQQCYHQAPT

CAPACASACEAQCPVVS

CEDACQTVCKGQCVFSGQNSRQ

CGPACAQSCSSLCHKKRVKRGEH

**ABU-6**

MRFTSLAIAFLACALAVSGSAIREKRQCGCAQPQQSQCSCQQVQQTQSCSCQSAPVQQQSPSCSCAQPQQTQQVQVQSTQ

CAPACQQSCQQQCQASPSVSQ

CQPQCQQQCQAQCTPMYNPPTTTTTTQAPVVQYQQ

CQPVCQQQCQSTCVQQQQPAAQ

CQPQCQQQCNVACDSPSTTTQAPQVIQIQLEIQQAQAQ

CQPQCQQQCQSSCVQQQQQSNQ

CEPACNTQCSDICQQTAQATQQVYNQNMNQNTNTQMYNPYNTNTSQNAN

CAPACQPACDNSCTSQQTQPMYQQYDTTTEAPAQVIQIVLQTSVAQSSQ

CAPQCEQSCQQQCVQQQQPAAQ

CQTACQSSCSNSCQTAQPATTACQQSPQQSSCSCQANYSPCGNGQCCRRK

**ABU-7**

MRFTSLAIAFLACALVVSGSVIREKRQCGCAQPQQSQCSCQQVQQTQSCSCQSAPVQQQSPSCSCAQPQQTQQVQVQSTQ

CAPACQQSCQQQCQASPSVSQ

CQPQCQQQCQAQCTPMYNPPTTTTTTQAPVVQYQQ

CQPVCQQQCQSTCVQQQQPAAQ

CQPQCQQQCNVACDSPSTTTQAPQVIQIQLEIQQAQAQ

CQPQCQQQCQSSCVQQQQPSTQ

CEPACNTQCSDICQQTAQATQQVYNQNMNQNTNTQMYNPYNTNTNQNAN

CAPACQPACDNSCTSQQAQPVYQQAQPTYQVQQTTAAPMYDPYNNQGSAN

CAPACQPACDNSCTSQQTQPMYQPYDTTTEAPAQVIQIVLQTSVAQSSQ

CAPQCEQSCQQQCVQQQQPAAQ

CQTACQSSCSNSCQTAQPATTACQQSPQQSSCSCQANYSPCGNGQCCRRK

**ABU-15**

MRFTSLSIAFLACALVVSGSAIREKRQCGCAQPQQSQCSCQQVQQTQSCSCQSAPVQQQSPSCSCAQPQQTQQVQVQSTQ

CAPACQQSCQQQCQASPSVSQ

CQPQCQQQCQAQCTPMYNPPTTTTTTQAPVVQYQQ

CQPVCQQQCQSTCVQQQQPAAQ

CQPQCQQQCNVACDSPSTTTQAPQVIQIQLEIQQAQAQ

CQPQCQQQCQSSCVQQQQQSNQ

CEPACNTQCSDICQQTAQATQQVYNQNMNQNTNTQMYNPYNTNTNQNAN

CAPACQPACDNSCTSQQTQPMYQPYDTTTEAPAQVIQIVLQTSVAQSSQ

CAPQCEQSCQQQCVQQQQPAAQ

CQTACQSSCSNSCQAAQPATTACQQSPQQSSCSCQANYSPCGNGQCCRRK

**ABU-8**

MRFTTLAVAFFACALVVSGSVLREKRQCGCAQPQQSQCSCQQVQQTQSCSCQSAPVQQQSPSCSCAQPQQTQQVQVQSTQ

CAPACQQSCQQQCQASPSVSQ

CQPQCQQQCQAQCTPMYNPPTTTTTTQAPVVQYQQ

CQPVCQQQCQSTCVQQQQPAAQ

CQPQCQQQCNVACDSPSTTTQAPQVIQIQLEIQQAQAQ

CQPQCQQQCQSSCVQQQQPSTQ

CEPACNTQCSDICQQTAQATQQVYNNNQNMNQNMNQNTNTQMYNPYNTNTNQGSAN

CAPACQPACDNSCTSQQAQPVYQQAQPTYQVQQTTAAPMYDPYNNQGSAN

CAPACQPACDNSCTSQQTQPMYQPYDTTTEAPAQVIQIVLQTSVAQSSQ

CAPQCEQSCQQQCVQQQQPAAQ

CQTACQSSCSNSCQAAQPATTACQQSPQQSSCSCQTNYSPCGNGQCCRRK

**M02G9.2**

MRSSWILVAFFVIASVQAIPQRTKRQNCDCTPGEAPKCGCQVMPTPEIGGGQMICTCSPPVPPKCVCTEGNVRNIITGPS

LPALFKPYN

CVSTCESSCLESCNRLNFNMK

CSSVCSEACEFTCAKQGTSTTPTTTQAPATTAMVPLPSNIQEYRFIIPAGSMLTKPTTSTSAPVILSQKIQIT

CPTECQPACSFVCTKLRPTLKVITQMFDSKTDETT

CRTSCSSACLSVCASSGVPGNE

CQSNCAPACEDTCTIVKPVAVATTTQIQSTSSSGGQ

CVSVCMPACTQQCMFTVQMQIALSQAVPQTTEKPTQAVLIPIAPVSIVVSDPPTTTAAPTVITLGTI

CRNECLVQCEHQCLPNNPNCMAACQTTCYPVCQSKMRRRMKETKNQRTEDFAHRQ

**PQN-54**

MRFTSLAIAFFACALVVSGNVLREKRQCGCAQPQQSQCSCQQVQQTQSCSCQSAPVQQQSPSCSCAQPQQTQNVQVQTTQ

CAPACQQSCTQQCQAAPSVAQ

CQPQCQQQCQSQCAPMYNPPATTTPAPVVQ

CQPMCQQQCQSTCVQQQQPASQ

CQPQCQQQCNVACDAPATSTQAPQVVHIQLEIQQAQAQ

CQPQCQQQCQSSCTQQQQPANQ

CNSACNSQCSNICQQTAQATQQVYNQNSNTNTQMYNPYNNNNQGSGSAN

CAPACQPACDNSCTSQTPAPMYNPYDASTSAPASSQAVIQIVLQSSVAQSSQ

CTPQCEQSCQQQCVQQQQPAAQ

CQSACQDSCSSSCQAAQPATTACQQSPQSPQNSCSCQANYSPCGNGQCCRRK

**PQN-57**
MRFITLAVFFACALVASSSVLREKRHCGCAQPQQSQCSCQQVQQTQSCSCQSAPVQQQAPSCSCAQPQQTQTVQVQSTQ

CAPACQQSCRQQCQSAPAVSQ

CQPMCQQQCQSQCTPMYNPPATTTTTPAPVVQ

CQPMCQQQCQSTCVQQQQPVSQ

CQPQCQQQCNVACDATTTTTSAPQVIHIQLEIQQAQVQ

CQPACQQQCQSSCVQQQQPAKQ

CASSCNTQCTNACQQTAQATQQVIYGQNSNTQMYDPYNNQQQQQAN

CAPACQPACDNSCIQQTAAPIYNPTTTSAPQVVQIVLQASVAQSSQ

CAPQCEQSCQQQCVQQQQPVAQ

CQSACQSSCSSSCQAAQPATVACQQAPQSNQCSCQSNYSPCGQGQCCRRK

**ABU-9**

MRFITLAVFFACALVASSSVLREKRHCGCAQPQQSQCSCQQVQQTQSCSCQSAPVQQQAPSCSCAQPQQTQTVQVQSTQ

CAPACQQSCRQQCQSAPAVSQ

CQPMCQQQCQSQCTPMYNPPATTTTTPAPVVQ

CQPMCQQQCQSTCVQQQQPVSQ

CQPQCQQQCNVACDATTTTTSAPQVIHIQLEIQQAQVQ

CQPACQQQCQSSCVQQQQPAKQ

CASSCNTQCTNACQQTAQATQQVIYGQNSNTQMYDPYNNQQQQQAN

CAPACQPACDNSCIQQTAAPIYNPTTTSAPQVVQIVLQASVAQSSQ

CAPQCEQSCQQQCVQQQQPVAQ

CQSACQSSCSSSCQAAQPATVACQQAPQSNQCSCQSNYSPCGQGQCCRRK

**M02G9.3**

MRCSIMWTFVLLAIPMVTSELFALTPVSRVRRQCCGRSSSSCCSSSSSNSY

CIPVCMAQCQSSCTTPI

CQQQCSNQCNQQCTSITISSGPS

CSSCQSACSSACTTPT

CIRTCQRNSCSNLCNTGSNS

CTNRCNSQCLQICTTPS

CTNTCSNSCSNACSNGGNQPIVIVIPSSSRN

CQNSCQNQCSSACTTMT

CRQTCQNTCLGSCNSCSGGGCNSNNSNLVVVTP

CERSCNSGCRSTCSSTSTLSV

CIPACQQTCRSTCSTAKTFVIPCTSGTSGNSCSCSTGYSICGSQCCRS

**ABU-14**

MAARRAASILLIAVLALSSTFVFADEETVKNETGLALSRAKRQCCSSNSNSCCGNNNNVQCIPVCLQQCQSS

CQTSQCIQQCQPACNQQCGGNNQVILLPQTNN

CNQCQQQCISSCATPI

CAQSCNNQCSSSCGNSAPQIVVLQPQQNQ

CGSCQSSCQQTCPT

CNCQSACAPACGGNNNNQQIIVVQQDNS

CSSNCNNQCSSSCSTPI

CIQSCQSSCQQACQPT

CQPQCMPSCSSSCTSNQAPIVIVAQQGDS

CSNSCSNQCQSACPTPI

CVQQCNSQCQSQCSNSCSGNSCNQQQVIVLQQQDN

CQNQCQSSCMNTCQSSATVLQ

CQPICQQTCQNTCQQAAQIVVPCQSSSSGCGCSSGYSQCGGSCCRRR

**ABU-11**

MNTKFSILFSLLAVSALAVAAEENVFLRARRQSSCGCAIPAAPQCNCQPAQTNTAQQSCSCAQAQQPSSCGCAQQYQQQQ

CAPTCQSSCEQSCVAQQQPIAQ

CQSSCSSTCQSACAQPVQLQQPAQQQ

CQQDCQAACPQQQQPQQQ

CQQQCQTTCQSDDQYSQQLIQQQTYAQPGQIPAYTGRSEYNTAPPQQQQASQ

CNTCQNSCLNTCQQQQQPVQQ

CQSSCDQSCQPQCATPSGSAYNQVNYQPQAQNNYQTNNNYQQPGAIPSYQGASQTAAPTYQPYRDTNVQVGGTISQ

CNTCQTQCNQECQVQQTPVNQ

CAPQCDAQCQPACSPAAPAAPVQTQQQIQLTINVPVARQSPQ

CQPQCEQSCNTQCIQQQQPISQCQPACQQSCSASCRK

**PQN-13,isoform a**
MVRWQPLLFALLVAFAYAADGTTKSEASSTTVATSESTTESTKTTTASAIKETVKEEVKEKEGPETRNETAAVSRAKRQC
CFAAQNSCCPQQVQSQCNCAALQIQVQQCDCANMLQTQCGCPSISSPSCDCGTLQNQYSSCGCGSLSYQVSCNQCQQQSQ
PQIIVVQQPQQQQ

CSSQCMPACLPSCVQSS

CAPACQPMCSSSCVQQQQQQIIVVQQPQQQ

CASSCMPSCQPSCVQQA

CAPACQPMCSSQCVEQQQAQIVVVQQPQQQ

CTSSCMPACQSSCVQQA

CAPACQPKCSSQCVEQQQAQIVVVQPPTSSSNN

CASSCMPQCTPQCVQQQTI

CAAACQPSCQSSCSSNAQ

CVQACLPSCESSCVQQQQPSVVVVQDSQSS

CPSACQPSCSQQCIQAQNL

CQSACQPSCQSSCGSNTQ

CVQACIPSCQQTCGQQAQPVIVVQQPQQNN

CVQACQPQCQQTCGSNVQ

CVSACQNSCQQSCGNQQQQQVIVVQSSPM

CGQACQAPPVLQCVPQCQPSCQPSCMQQYQFIQAPAITMCQQSGGSCGCGSGYSQCMQGVCCLRKRHRLAKNKL

**F35A5.4**

MQYLIAAFAVFQLSLGDEFSTSALTSPYLKMNGSLITRVRSKRQCCAMCPSGNCGCGSCSGFVT

CPDRCQPMCTQACVMAKTSV

CVDQCMPKCDSACINLVRSGPS

CDQQCMPLCLPACINAIQGPTE

CAPQCMPSCSSNCIQQVFPS

CPQQCQPVCTPQCIQSIQVAIQRPT

CASSCMPSCSQSCIQKYEITVEQET

CVPACMPACSSACVQAVT

CSTCTNNCPSICSQAN

CIPQCMPRCLPTCIQQIQISVPLPPPAPR

CDSMCMPSCSPSCVQQYSL

CPQQCRPMCTNACVTNLQVSRPVSQ

CLPACMPSCTSSCVQSM

CPQQCYPSCDMNCIQQYAVQMAPTP

CAAQCMPLCESSCIARLS

CVQTCMPACSPTCVQNYAAGPIQ

CAPACMPLCQPTCVQQHAEVVVACGVPCQCQPGYVQCSQNLCCLKYKNMAAKFRKLSGSTTNSNNNGNNGHNNGPNNNNDEDDEDNSAGGSYTEPMRDSPKTSKSNSTSSEEKNSGKTLSAFSDTQMDGDSYAKA
